# Effect of CLU genetic variants on cerebrospinal fluid and neuroimaging markers in healthy, mild cognitive impairment and Alzheimer’s disease cohorts

**DOI:** 10.1038/srep26027

**Published:** 2016-05-27

**Authors:** Lin Tan, Hui-Fu Wang, Meng-Shan Tan, Chen-Chen Tan, Xi-Chen Zhu, Dan Miao, Wan-Jiang Yu, Teng Jiang, Lan Tan, Jin-Tai Yu, Alzheimer’s Disease Neuroimaging Initiative, Michael W. Weiner, Paul Aisen, Ronald Petersen, Clifford R. Jack, William Jagust, John Q. Trojanowki, Arthur W. Toga, Laurel Beckett, Robert C. Green, Andrew J. Saykin, John Morris, Leslie M. Shaw, Jeffrey Kaye, Joseph Quinn, Lisa Silbert, Betty Lind, Raina Carter, Sara Dolen, Lon S. Schneider, Sonia Pawluczyk, Mauricio Beccera, Liberty Teodoro, Bryan M. Spann, James Brewer, Helen Vanderswag, Adam Fleisher, Judith L. Heidebrink, Joanne L. Lord, Sara S. Mason, Colleen S. Albers, David Knopman, Kris Johnson, Rachelle S. Doody, Javier Villanueva-Meyer, Munir Chowdhury, Susan Rountree, Mimi Dang, Yaakov Stern, Lawrence S. Honig, Karen L. Bell, Beau Ances, John C. Morris, Maria Carroll, Mary L. Creech, Erin Franklin, Mark A. Mintun, Stacy Schneider, Angela Oliver, Daniel Marson, Randall Griffith, David Clark, David Geldmacher, John Brockington, Erik Roberson, Marissa Natelson Love, Hillel Grossman, Effie Mitsis, Raj C. Shah, Leyla deToledo-Morrell, Ranjan Duara, Daniel Varon, Maria T. Greig, Peggy Roberts, Marilyn Albert, Chiadi Onyike, Daniel D’Agostino, Stephanie Kielb, James E. Galvin, Brittany Cerbone, Christina A. Michel, Dana M. Pogorelec, Henry Rusinek, Mony J. de Leon, Lidia Glodzik, Susan De Santi, P. Murali Doraiswamy, Jeffrey R. Petrella, Salvador Borges-Neto, Terence Z. Wong, Edward Coleman, Charles D. Smith, Greg Jicha, Peter Hardy, Partha Sinha, Elizabeth Oates, Gary Conrad, Anton P. Porsteinsson, Bonnie S. Goldstein, Kim Martin, Kelly M. Makino, M. Saleem Ismail, Connie Brand, Ruth A. Mulnard, Gaby Thai, Catherine Mc-Adams-Ortiz, Kyle Womack, Dana Mathews, Mary Quiceno, Allan I. Levey, James J. Lah, Janet S. Cellar, Jeffrey M. Burns, Russell H. Swerdlow, William M. Brooks, Liana Apostolova, Kathleen Tingus, Ellen Woo, Daniel H. S. Silverman, Po H. Lu, George Bartzokis, Neill R. Graff-Radford, Francine Parfitt, Tracy Kendall, Heather Johnson, Martin R. Farlow, Ann Marie Hake, Brandy R. Matthews, Jared R. Brosch, Scott Herring, Cynthia Hunt, Christopher H. van Dyck, Richard E. Carson, Martha G. MacAvoy, Pradeep Varma, Howard Chertkow, Howard Bergman, Chris Hosein, Sandra Black, Bojana Stefanovic, Curtis Caldwell, Ging-Yuek Robin Hsiung, Howard Feldman, Benita Mudge, Michele Assaly, Elizabeth Finger, Stephen Pasternack, Irina Rachisky, Dick Trost, Andrew Kertesz, Charles Bernick, Donna Munic, Marek-Marsel Mesulam, Kristine Lipowski, Sandra Weintraub, Borna Bonakdarpour, Diana Kerwin, Chuang-Kuo Wu, Nancy Johnson, Carl Sadowsky, Teresa Villena, Raymond Scott Turner, Kathleen Johnson, Brigid Reynolds, Reisa A. Sperling, Keith A. Johnson, Gad Marshall, Jerome Yesavage, Joy L. Taylor, Barton Lane, Allyson Rosen, Jared Tinklenberg, Marwan N. Sabbagh, Christine M. Belden, Sandra A. Jacobson, Sherye A. Sirrel, Neil Kowall, Ronald Killiany, Andrew E. Budson, Alexander Norbash, Patricia Lynn Johnson, Thomas O. Obisesan, Saba Wolday, Joanne Allard, Alan Lerner, Paula Ogrocki, Curtis Tatsuoka, Parianne Fatica, Evan Fletcher, Pauline Maillard, John Olichney, Charles DeCarli, Owen Carmichael, Smita Kittur, Michael Borrie, T -Y Lee, Rob Bartha, Sterling Johnson, Sanjay Asthana, Cynthia M. Carlsson, Steven G. Potkin, Adrian Preda, Dana Nguyen, Pierre Tariot, Anna Burke, Nadira Trncic, Adam Fleisher, Stephanie Reeder, Vernice Bates, Horacio Capote, Michelle Rainka, Douglas W. Scharre, Maria Kataki, Anahita Adeli, Earl A. Zimmerman, Dzintra Celmins, Alice D. Brown, Godfrey D. Pearlson, Karen Blank, Karen Anderson, Laura A. Flashman, Marc Seltzer, Mary L. Hynes, Robert B. Santulli, Kaycee M. Sink, Leslie Gordineer, Jeff D. Williamson, Pradeep Garg, Franklin Watkins, Brian R. Ott, Henry Querfurth, Geoffrey Tremont, Stephen Salloway, Paul Malloy, Stephen Correia, Howard J. Rosen, Bruce L. Miller, David Perry, Jacobo Mintzer, Kenneth Spicer, David Bachman, Nunzio Pomara, Raymundo Hernando, Antero Sarrael, Norman Relkin, Gloria Chaing, Michael Lin, Lisa Ravdin, Amanda Smith, Balebail Ashok Raj, Kristin Fargher

**Affiliations:** 1 College of Medicine and Pharmaceutics, Ocean University of China, China; 2 Department of Neurology, Qingdao Municipal Hospital, Nanjing Medical University, Qingdao, China; 3 Department of Neurology, Qingdao Municipal Hospital, School of Medicine, Qingdao University, Qingdao, China; 4 Department of Radiology, Qingdao Municipal Hospital, School of Medicine, Qingdao University, Qingdao, China; 5 Department of Neurology, Nanjing First Hospital, Nanjing Medical University, Nanjing, China; 6 Magnetic Resonance Unit at the VA Medical Center and Radiology, Medicine, Psychiatry and Neurology, University of California, San Francisco, USA; 7 San Diego School of Medicine, University of California, California, USA; 8 Mayo Clinic, Minnesota, USA; 9 Mayo Clinic, Rochester, USA; 10 University of California, Berkeley, USA; 11 University of Pennsylvania, Pennsylvania, USA; 12 University of Southern California, California, USA; 13 University of California, Davis, California, USA; 14 MPH Brigham and Women’s Hospital/Harvard Medical School Massachusetts, USA; 15 Indiana University, Indiana, USA; 16 Washington University St. Louis, Missouri, USA; 17 Oregon Health and Science University, Oregon, USA; 18 University of California--San Diego, California, USA; 19 University of Michigan, Michigan, USA; 20 Baylor College of Medicine, Houston, State of Texas, USA; 21 Columbia University Medical Center, South Carolina, USA; 22 University of Alabama – Birmingham, Alabama, USA; 23 Mount Sinai School of Medicine, New York, USA; 24 Rush University Medical Center, Rush University, Illinois, USA; 25 Wien Center, Florida, USA; 26 Johns Hopkins University, Maryland, USA; 27 New York University, NY, USA; 28 Duke University Medical Center, North Carolina, USA; 29 University of Kentucky, Kentucky, USA; 30 University of Rochester Medical Center, NY, USA; 31 University of California, Irvine, California, USA; 32 University of Texas Southwestern Medical School, Texas, USA; 33 Emory University, Georgia, USA; 34 University of Kansas, Medical Center, Kansas, USA; 35 University of California, Los Angeles, California, USA; 36 Mayo Clinic, Jacksonville, USA; 37 Yale University School of Medicine, Connecticut, USA; 38 McGill University, Montreal-Jewish General Hospital, Canada; 39 Sunnybrook Health Sciences, Ontario, USA; 40 U.B.C. Clinic for AD & Related Disorders, Canada; 41 Cognitive Neurology - St. Joseph’s, Ontario, USA; 42 Cleveland Clinic Lou Ruvo Center for Brain Health, Ohio, USA; 43 Northwestern University, USA; 44 Premiere Research Inst (Palm Beach Neurology), USA; 45 Georgetown University Medical Center, Washington D.C, USA; 46 Brigham and Women’s Hospital, Massachusetts, USA; 47 Stanford University, California, USA; 48 Banner Sun Health Research Institute, USA; 49 Boston University, Massachusetts, USA; 50 Howard University, Washington D.C, USA; 51 Case Western Reserve University, Ohio, USA; 52 University of California, Davis – Sacramento, California, USA; 53 Neurological Care of CNY, USA; 54 Parkwood Hospital, Pennsylvania, USA; 55 University of Wisconsin, Wisconsin, USA; 56 University of California, Irvine – BIC, USA; 57 Banner Alzheimer’s Institute, USA; 58 Dent Neurologic Institute, NY, USA; 59 Ohio State University, Ohio, USA; 60 Albany Medical College, NY, USA; 61 Hartford Hospital, Olin Neuropsychiatry Research Center, Connecticut, USA; 62 Dartmouth-Hitchcock Medical Center, New Hampshire, USA; 63 Wake Forest University Health Sciences, North Carolina, USA; 64 Rhode Island Hospital, state of Rhode Island, USA; 65 Butler Hospital, Providence, Rhode Island, USA; 66 University of California, San Francisco, USA; 67 Medical University South Carolina, USA; 68 Nathan Kline Institute, Orangeburg, New York, USA; 69 Cornell University, Ithaca, New York, USA; 70 USF Health Byrd Alzheimer’s Institute, University of South Florida, USA

## Abstract

The Clusterin (*CLU*) gene, also known as apolipoprotein J (*ApoJ*), is currently the third most associated late-onset Alzheimer’s disease (LOAD) risk gene. However, little was known about the possible effect of *CLU* genetic variants on AD pathology in brain. Here, we evaluated the interaction between 7 *CLU* SNPs (covering 95% of genetic variations) and the role of *CLU* in β-amyloid (Aβ) deposition, AD-related structure atrophy, abnormal glucose metabolism on neuroimaging and CSF markers to clarify the possible approach by that *CLU* impacts AD. Finally, four loci (rs11136000, rs1532278, rs2279590, rs7982) showed significant associations with the Aβ deposition at the baseline level while genotypes of rs9331888 (P = 0.042) increased Aβ deposition. Besides, rs9331888 was significantly associated with baseline volume of left hippocampus (P = 0.014). We then further validated the association with Aβ deposition in the AD, mild cognitive impairment (MCI), normal control (NC) sub-groups. The results in sub-groups confirmed the association between *CLU* genotypes and Aβ deposition further. Our findings revealed that *CLU* genotypes could probably modulate the cerebral the Aβ loads on imaging and volume of hippocampus. These findings raise the possibility that the biological effects of *CLU* may be relatively confined to neuroimaging trait and hence may offer clues to AD.

Alzheimer’s disease (AD) is the most common form of dementia in the elderly, accounting for 50% of all dementia^1^. It has been documented that genetic factors, along with environments, extremely contributes to the pathogenesis of AD^2^,^3^. Clusterin gene (*CLU*), also known as apolipoprotein J (*ApoJ*), is currently the third most associated risk gene according to Alzgene database (http://www.alzgene.org/). It is located in chromosome 8p21–p12 which is a chromosomal region of interest in AD^4^ and it may explain around 9% of the late-onset AD (LOAD) attributable risk^5^,^6^. Many large genome-wide association studies (GWAS) have identified that rs2279590, rs11136000, rs9331888, rs7012010, rs7982 and rs1532278 in *CLU* was substantially associated with AD risk in individuals of Caucasian ancestry and other populations^7^,^8^,^9^,^10^,^11^. Several independent candidate gene studies have then replicated and confirmed these results in various Caucasian populations or other populations, although the strongest associated variant sometimes differed^12^,^13^,^14^,^15^,^16^,^17^,^18^,^19^,^20^,^21^,^22^,^23^. Our group previously reported that rs9331949 and rs9331888 variation in the *CLU* gene played significant role in sporadic LOAD in the Han Chinese population^24^,^25^,^26^,^27^.

Regarding to the mechanisms how the *CLU* gene polymorphism induce the risk for AD, efforts to identify functional variations through exon sequencing and examining effects of SNPs on *CLU* expression in brain tissue have not yet provided a functional link between the associated polymorphisms and AD^28^, such as is seen in *ApoE*^29^. To date, the risk allele of the AD-associated SNP rs9331888, associated with the alternative splicing of *CLU* gene^30^, increases the relative abundance of transcript NM_203339. Coincidently, the results of our previous study also revealed that the AD risk rs9331888 allele was associated with a decrease in *CLU* plasma levels^27^. Another risk allele of the AD-associated SNP rs11136000 was significantly associated with lower clusterin plasma levels in an allele-dose-dependent manner^31^,^32^. It also modified CSF levels of the microtubule-associated protein tau and decreased Aβ(1-42) in AD patients^33^,^34^. For more than two decades, the “amyloid hypothesis” has been the leading scientific explanation for AD^35^. Convincing evidence suggests that the physical interaction of clusterin with amyloid β (Aβ) plays an important role in AD pathogenesis^28^. Briefly, these evidences supported that *CLU* polymorphisms could modulate AD susceptibility by altering Aβ accumulation in the current literature. To date, as florbetapir 18F amyloid PET and CSF Aβ1-42 are reported to reflect the brain amyloid burden with high specificity^36^,^37^, multiple neuroimaging measures, along with CSF proteins (Aβ1-42 and tau) could be proposed as critical markers in biological research and clinical trials in AD pathophysiological process^38^. Intriguingly, these neuroimaging methods are likely to be shaped by genetic influences with heritability^39^.

From the above evidence, it is possible that *CLU* genetic variations mediate the susceptibility of AD by altering the biomarkers of Aβ accumulation (including low Aβ42 in CSF and abnormal Aβ deposition on imaging) and the neuronal degeneration biomarkers. The evidence that AD susceptible gene could affect neuroimaging and CSF markers would further confirm the roles of these genetic factors in AD. To ascertain whether *CLU* polymorphisms mediate the susceptibility of AD by altering the biomarkers of Aβ accumulation and neuronal degeneration biomarkers, we genotyped *CLU* polymorphisms and explored their associations with AD specific brain structures and functions on imaging and CSF to investigate the mechanism.

## Results

### Demographics

The dataset comprised of 812 individuals, including 281 normal controls (NC), 483 mild cognitive impairment (MCI) and 48 AD at baseline. The demographics and the clinical data were summarized in Supplementary Table S1 while the SNP distributions were in Table 1. No statistical differences were observed among NC, MCI and AD patients when comparing the distribution of all the tested SNPs allele frequencies in our study.

### Impacts of *CLU* genotypes on Aβ deposition

**Figure 1:**
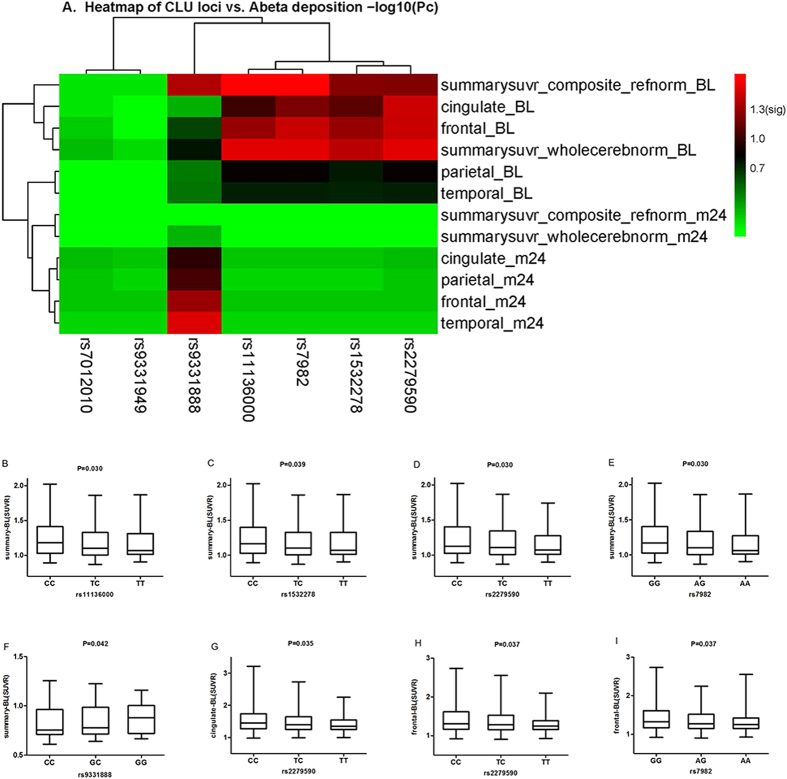
The correlation between *CLU* genetic variants and Aβ accumulation on AV45. (**A**) Heatmap of correlation between *CLU* genetic variants and Aβ accumulation on AV45. The statistical relations (FDR-corrected P values) between Aβ accumulation on AV45 (rows) and *CLU* loci (columns) (**B**) rs11136000 was associated with the level of summary SUVR at baseline. The X-axis represents three genotypes while the Y-axis represents the summary AV45 retention at baseline. (**C**) rs1532278 was associated with the level of summary SUVR at baseline. The X-axis represents three genotypes while the Y-axis represents the summary AV45 retention at baseline. (**D**) rs2279590 was associated with the level of summary SUVR at baseline. The X-axis represents three genotypes while the Y-axis represents the summary AV45 retention at baseline. (**E**) rs7982 was associated with the level of summary SUVR at baseline. The X-axis represents three genotypes while the Y-axis represents the summary AV45 retention at baseline. (**F**) rs9331888 was associated with the level of summary SUVR at baseline. The X-axis represents three genotypes while the Y-axis represents the summary AV45 retention at baseline. (**G**) rs2279590 was associated with the level of cingulate SUVR at baseline. The X-axis represents three genotypes while the Y-axis represents the cingulate AV45 retention at baseline. (**H**) rs2279590 was associated with the level of frontal cortex SUVR at baseline. The X-axis represents three genotypes while the Y-axis represents the frontal AV45 retention at baseline. (**I**) rs7982 was associated with the level of frontal cortex SUVR at baseline. The X-axis represents three genotypes while the Y-axis represents the frontal AV45 retention at baseline. Note: SUVR, standard uptake value ratios; AV45, amyloid tracer.

In this study we compared the levels of tracer retention in frontal, parietal, temporal cortex and cingulate, as well as summary florbetapir standard uptake value ratios (SUVRs) among three different allelotypes in each locus at baseline. We analyzed them in the whole group and then validated significant loci in the three different clinical stages (AD, MCI, and NC). The AV-45 retention on amyloid PET imaging represented the Aβ deposition. Thus we measured Aβ deposition in brain to test the relationships between *CLU* genotypes and levels of tracer retention on amyloid PET imaging. We investigated the relationship between the Aβ deposition and the seven loci in multiple linear regression analysis (Fig. 1A, Supplementary Table S2 and S3). Finally, four loci (rs11136000, rs1532278, rs2279590, rs7982) showed significant associations with the Aβ deposition at the baseline level of all the subjects (Table 2). Among the SNPs, three genotypes of rs11136000 (P = 0.030) (Fig. 1B), rs1532278 (P = 0.039) (Fig. 1C), rs2279590 (P = 0.030) (Fig. 1D) and rs7982 (P = 0.030) (Fig. 1E) were significantly associated with tracer retention in summary SUVR while genotypes of rs9331888 (P = 0.042) increased tracer retention in summary SUVR (Fig. 1F). Besides, three genotypes of rs2279590 decreased tracer retention in cingulate (P = 0.035) (Fig. 1G) and frontal cortex (P = 0.037) (Fig. 1H). Three genotypes of rs7982 decreased tracer retention in frontal cortex (P = 0.037) as well (Fig. 1I). Moreover, we performed linkage disequilibrium (LD) analysis and discovered that rs7982, rs11136000, rs1532278 and rs9331888 were in LD (Supplementary Figure S1). In the haplotype-based analysis, the haplotypes (GCCG, ATTC) were observed to be related to the levels of amyloid deposition (P < 0.05) and this supported that CLU modulates the alteration of the biomarkers of Aβ markers to influence the risk of AD *in vivo* (Supplementary Table S15).

**Figure 2:**
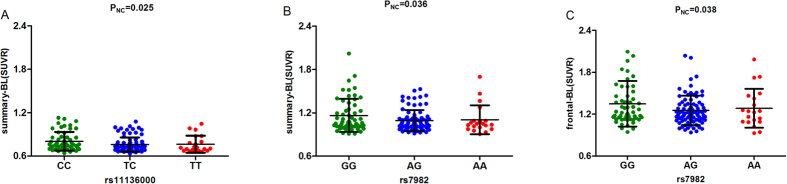
The correlation between significant loci and AV45 SUVR at baseline in NC group. (**A**) rs11136000 was associated with the level of summary SUVR at baseline. The X-axis represents three genotypes while the Y-axis represents the summary AV45 retention at baseline. (**B**) rs7982 was associated with the level of summary SUVR at baseline. The X-axis represents three genotypes while the Y-axis represents the summary AV45 retention at baseline. (**C**) rs7982 was associated with the level of frontal cortex SUVR at baseline. The X-axis represents three genotypes while the Y-axis represents the frontal AV45 retention at baseline. Note: SUVR, standard uptake value ratios; AV45, amyloid tracer.

**Figure 3:**
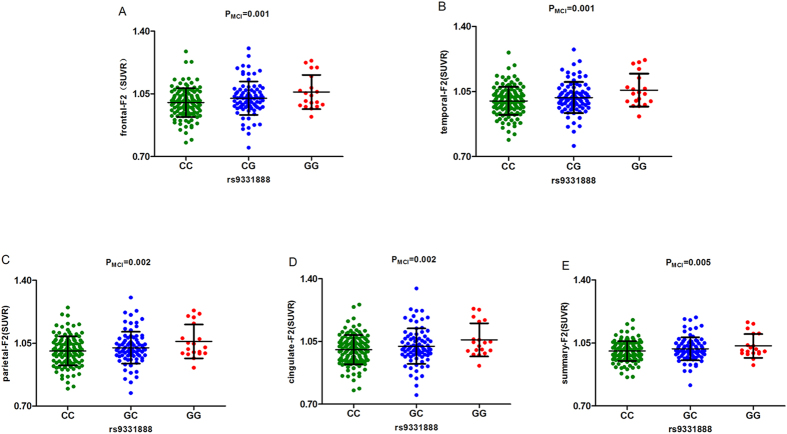
The correlation between rs9331888 and AV45 SUVR in two-year follow-up study in MCI group. (**A**) rs9331888 was associated with the level of frontal cortex SUVR at two-year follow study. The X-axis represents three genotypes while the Y-axis represents the frontal AV45 retention. (**B**) rs9331888 was associated with the level of temporal cortex SUVR at two-year follow study. The X-axis represents three genotypes while the Y-axis represents the temporal AV45 retention. (**C**) rs9331888 was associated with the level of parietal cortex SUVR at two-year follow study. The X-axis represents three genotypes while the Y-axis represents the parietal AV45 retention. (**D**) rs9331888 was associated with the level of cingulate SUVR at two-year follow study. The X-axis represents three genotypes while the Y-axis represents the cingulate AV45 retention. (**E**) rs9331888 was associated with the level of summary SUVR at two-year follow study. The X-axis represents three genotypes while the Y-axis represents the summary AV45 retention. Note: SUVR, standard uptake value ratios; AV45, amyloid tracer.

We then further validated the above results in the AD, MCI, NC sub-groups. In the NC group, rs11136000 and rs7982 were found to be significant. Three genotypes of rs11136000 (P = 0.025) (Fig. 2A) and rs7982 (P = 0.036) (Fig. 2B) were validated to decrease the tracer retention in summary SUVR at baseline. Rs7982 also decreased tracer retention in frontal cortex at baseline (P = 0.038) (Fig. 2C). In the MCI group, rs9331888 was the only loci found to be significant in two-year follow-up study. It increased the tracer retention in frontal (P = 0.001), parietal (P = 0.002), temporal cortex (P = 0.001) and cingulate (P = 0.002), as well as summary SUVR (P = 0.005) among three different allelotypes (Fig. 3). In the AD group, none of the above loci were validated to be significant.

### Impacts of *CLU* genotypes on MRI structure

We analyzed the association of these *CLU* loci with AD related brain structures (middle temporal gyrus, posterior cingulate, precuneus, parahippocampal gyrus and hippocampus, as well as the thickness of entorhinal cortex)^40^,^41^,^42^,^43^ in a model which rectified age, gender, education years, ApoE ε4 status and intracranial volume (ICV) as covariates at baseline and two-year followup study (Supplementary Table S4–S11). In the whole group, only single nucleotide polymorphisms (SNPs) at rs9331888 was significantly associated with baseline volume of left hippocampus (P = 0.014). As for the thichness of right entorhinal cortex, SNPs at rs9331888 was significant in the cross-section analysis in baseline (P = 0.016) and two-year follow-up study (P = 0.011) while rs11136000 was significant in the cross-section analysis in two-year follow-up study (P = 0.042), but none of the difference achieved the significant level after the FDR correction. However, none of the loci was significantly associated with hippocampal subfields volume of CA1 in the cross-section analysis or in a multiple linear regression model.

In the AD group, SNPs at rs9331888 were significantly associated with volume of left hippocampus (P = 0.004) in two-year follow-up study. However, in the MCI and NC group, the SNPs at rs9331888 were not significantly associated with volume of left hippocampus.

### Impacts of *CLU* genotypes on CSF markers

We firstly investigated the correlations between the concentrations of CSF proteins (Aβ, T-tau and P-tau) and *CLU* genotypes in a multiple linear regression model (Supplementary Table S12). We did not figure out any marked relationships between the levels of Aβ, T-tau, P-tau and these *CLU* genotypes at baseline. However, in the cross-section analysis, the levels of T-tau showed remarkable difference among the three genotypes of rs11136000 (P = 0.026), but none of the difference achieved the significant level in the FDR test. In a word, we did not detect any association between the *CLU* genetic variations and CSF markers.

### Impacts of *CLU* genotypes on glucose metabolism

In the analysis of the cerebral metabolism rate of glucose (CMRgl) on FDG-PET imaging, amygdala, posterior cingulate and temporal cortex were considered as targeted regions to detect their associations with *CLU* polymorphisms (Supplementary Table S13 and S14). We observed that the three genotypes at rs7012010 had different metabolism rate in left angular (P = 0.049) at baseline, but the significant difference lost after FDR correction (P = 0.34). As a result, we did not detect any association between the *CLU* genetic variations and glucose metabolism.

## Discussion

Our imaging-genetics analysis in ADNI dataset suggested that *CLU* genotypes impacted the Aβ deposition on amyloid PET imaging. Besides, rs9331888 polymorphism was still linked to the atrophy of hippocampus, especially in the AD patients. However, no evidence supported that *CLU* genotypes impacted CSF markers and FDG uptake on PET. These findings further disclosed that *CLU* might participate mainly in the Aβ deposition and hippocampus atrophy, leading to modulate the susceptibility of AD.

Our findings suggest that *CLU* variants that modulate AD risk may act through their influence on Aβ deposition and hippocampus atrophy. The possible mechanisms investigated in the current study were mostly consistent with the previous reports about the involvement of *CLU* in the pathogenesis of AD. Previous research reported that clusterin immunoreactivity is present in amyloid deposits, neuropil threads, dystrophic neurites in senile plaques, but is rarely observed in NFT-containing neurons^44^. Using PET imaging, it was also demonstrated that increased plasma clusterin concentrations were positively associated with fibrillar Aβ burden in the entorhinal cortex in AD patients^45^. In addition, In addition, animal studies from 10 years ago linking CLU/APOJ to amyloid deposition have shown that clusterin/Aβ interactions play an important role in amyloid formation and toxicity^46^,^47^. In the PDAPP mice, thioflavine-S-positive amyloid that deposits in the absence of clusterin was associated with far less neuritic dystrophy than amyloid present in clusterin-expressing PDAPP mice. Evidence also showed that the in vivo effects of clusterin on amyloid formation are likely to involve multiple interactions and processes in *ApoE*-negative PDAPP mice models^48^. These studies have provided evidence for a protective role of clusterin in AD pathogenesis, such as prevention of Aβ fibrillization, clearance of Aβ, inhibition of the complement system and neuronal apoptosis, and promotion of neurite outgrowth^49^,^50^,^51^,^52^. Coincidently, we also found that four loci (rs11136000, rs1532278, rs2279590, rs7982) were significantly associated with Aβ deposition in cingulate, frontal cortex and summary SUVR of brain. There was the least Aβ deposition in the homozygote mutant of the four loci (Fig. 1). For example, the subjects who carried the CC allele of rs11136000 had the most Aβ deposition than TC while those with TT allele had the least Aβ deposition (Fig. 1B). Furthermore, rs11136000 and rs7982 were certificated to be still protective in the NC group. Previously available evidence strongly supported the position that the initiating event in AD was related to abnormal processing of Aβ, ultimately leading to formation of Aβ plaques in the brain. This process occurs while individuals are still cognitively normal^53^. Our result also strongly indicated that conclusion. Notably, the homozygous mutant of rs11136000 and rs7982 acted as protective role in Aβ deposition in the NC group.

In our current study, rs9331888 plays an important role in Aβ deposition as well. It is widely recognized that the minor allele (G) of the rs9331888 polymorphism within *CLU* was previously reported to be significantly associated with an increased risk of LOAD^24^. The genotypes of rs9331888 in this study were associated with tracer retention in summary SUVR (Fig. 1F). In the MCI group, rs9331888 was the only loci found to be significant in two-year follow-up study. It increased the tracer retention in frontal (P = 0.001), parietal (P = 0.002), temporal cortex (P = 0.001) and cingulate (P = 0.002), as well as summary SUVR (P = 0.005) among three different alleles (Fig. 3). This means that the homozygous mutant (GG) of rs9331888 acted as a risk factor in Aβ deposition (Fig. 1F). In addition, the risk allele of the AD-associated SNP rs9331888, associated with the alternative splicing of *CLU* gene^30^, increases the relative abundance of transcript NM_203339. Coincidently, the results of our previous study also revealed that the AD risk rs9331888 allele was associated with a decrease in clusterin plasma level^27^. All the above indicated that it may work by increasing Aβ deposition during AD progression. As a result, evaluating the extent of AD pathology using rs9331888 in patients with MCI could provide clues regarding Aβ deposition underlying progression to AD and assist with early identification of patients with greatest risk to progress to an AD diagnosis, which will be important for clinical trials and treatment development.

Despite the risk in Aβ deposition, rs9331888 was also significantly associated with baseline volume of left hippocampus in the whole group. Genotypes in rs9331888 were further validated to be associated with volume of left hippocampus in two-year follow-up study in the AD group instead of the MCI and NC group. Patients carried with GG genotype showed a smaller volume of hippocampus, as well as the decline of cognition. This is coincident to a study on cognition by Mengel^54^. However, the impacts of *CLU* genotypes on MRI structure we discovered were not completely coincident with other studies. They found that clusterin levels have been correlated with symptom severity, entorhinal/hippocampal cortex atrophy, and Aβ burden^45^,^49^,^55^. The following reasons may explain the differences. Firstly, we genotyped 7 SNPs in *CLU*, while only one locus (rs11136000) were tested in previous study. Besides, we validated their correlations in the three different diagnosis groups respectively, which was also different from the previous study.

*CLU* has been demonstrated to be present in lipoprotein particles in CSF. Level of clusterin protein in CSF is significantly increased in AD patients^56^. Reports found that *CLU* rs11136000 SNP modified CSF levels of the microtubule-associated protein Tau and decreased Aβ (1-42) in AD patients^33^,^34^. However, other study denied this significance^12^,^57^. However, no evidence supported that *CLU* genotypes impact the Aβ burden or tau in CSF in our study. More evidence may be needed to explain the interactions between *CLU* and Aβ burden in CSF.

Genetically, multiple variations within *CLU*, such as rs2279590, rs11136000, rs9331888, rs7012010, rs9331949, rs7982 and rs1532278, have been identified to be associated with the risk of AD in multi-center, large scale GWAS, meta-analysis or replication studies. Among these loci, rs11136000 and rs9331888 were mostly investigated. Moreover, we performed linkage disequilibrium (LD) analysis and discovered that rs7982, rs11136000, rs1532278 and rs9331888 were in LD. The haplotypes (GCCG, ATTC) were related to the levels of amyloid deposition. Thus the haplotype-based analysis validated that *CLU* genotypes were related to the levels of amyloid deposition. The results presented here are not only correlative, but also support that *CLU* modulates the alteration of the biomarkers of Aβ markers to influence the risk of AD *in vivo*.

To date, continuous variable phenotypic analysis is now widely used to elucidate the specific role of genetics of multiple diseases. Distincted from the previous two categorical variable analysis (case *vs* control), the phenotypic analysis can not only be more sensitive to the association between genetic mutation and AD, but also provide more intuitively to explain the specific genetic effects on brain structure and function^58^. To date, numbers of GWAS–validated or GWAS-promising candidate loci have been certificated that they influence imaging and clinical features in AD^40^,^59^,^60^,^61^.

The advantage of our study is the method we use. Imaging genetics is an emergent transdisciplinary research field, in which genetic risk is assessed with imaging measures as quantitative traits (QTs) or continuous phenotypes. QT association studies have increased statistical power and decreased sample size requirements, thus imaging genetics studies have advantages over traditional case-control designs^62^,^63^. Although the differences across phenotypes with the same SNP might reflect power differences due to sample size differences, our findings that *CLU* modulates the alteration of the biomarkers of Aβ markers to influence the risk of AD in vivo were also supported that by animal studies from 10 years ago linking CLU/APOJ to amyloid deposition. Hence, the important role of this paper is that it confirmed the results of animal studies with in vivo neuroimaging data. However, the neuroimaging data were available only in a subset of participants in some QT analyses, e.g., half of participants with MRI information, 70% with FDG-PET, and 55% with AV45. Therefore, the QT analysis had a reduced sample size in some cases. Besides, the ADNI data was restricted to Caucasians to avoid genetics stratification across ethnicities. The 7 loci in *CLU*, however, have different frequencies in different races; therefore, our results cannot represent the other ethnicities, warranting the replications in other races.

In summary, our results showed that four loci (rs11136000, rs1532278, rs2279590, rs7982) showed significant associations with the Aβ deposition at the baseline level while genotypes of rs9331888 (P = 0.042) increased Aβ deposition. Besides, rs9331888 was significantly associated with baseline volume of left hippocampus (P = 0.014). We then further validated the association with Aβ deposition in the AD, mild cognitive impairment (MCI), normal control (NC) sub-groups. The results in sub-groups confirmed the association between *CLU* genotypes and Aβ deposition further. Moreover, our findings are also supported by animal studies from 10 years ago linking CLU/APOJ to amyloid deposition. These findings further supported the hypothesis that *CLU* genetic variations modulate the alteration of the biomarkers of Aβ markers to influence the risk of AD. These findings raise the possibility that the biological effects of *CLU* may be relatively confined to neuroimaging trait and hence may offer clues to the mechanisms through which particular genetic variants might influence AD risk.

## Methods

### ADNI dataset

The data in this study were obtained from Alzheimer’s Disease Neuroimaging Initiative (ADNI)^64^. ADNI is a large, multicenter, longitudinal neuroimaging study, launched in 2003 by the National Institute on Aging, the National Institute of Biomedical Imaging and Bioengineering, the Food and Drug Administration, private pharmaceutical companies, and nonprofit organizations^65^. The initial goal of ADNI is to recruit 800 subjects. However, it has been followed by ADNI-GO and ADNI-2. Thus these three protocols have covered more than 1500 adults who are 55 to 90 years old to participate in the research, including cognitively normal (CN) older individuals, mild cognitive impairment (MCI), and early dementia patients with due to AD^66^. The study was approved by the institutional review boards of all participating centers (Ocean University of China, Qingdao Municipal Hospital, Nanjing First Hospital, Memory and Aging Center in University of California, and ADNI) and written informed consent was obtained from all participants or authorized representatives. In addition, the methods were carried out in accordance with the approved guidelines.

### Participants

Participants were screened and enrolled according to criteria demonstrated in the ADNI study protocol (http://www.adni-info.org/scientists/adnistudyprocedures.aspx). We restricted the participants to whose genotype data of *CLU* SNPs were available and comprised 812 individuals. Baseline and longitudinal data including structural MRI and PET results were collected and all participants underwent a battery of clinical tests including Clinical Dementia Rating scale sum of boxes (CDRSB), Alzheimer’s disease Assessment Scale (ADAS-cog), Mini-Mental State Exam (MMSE), Rey Auditory Verbal Learning Test (RAVLT) and Functional Activities Questionnaire (FAQ) at baseline. According to the National Institute of Neurological and Communication Disorders/Alzheimer’s Disease and Related Disorders Association criteria for probable AD (NINCDS-ADRDA: probable AD), participants of AD were included if with a MMSE score between 20 and 26, a global Clinical Dementia Rating (CDR) of 0.5 or 1.0 and a CDRSB of 1.0 to 9.0. Amnestic MCI subjects achieved a MMSE score of 24 to 30 as well as a CDR score of 0.5 while the cognitively normal control individuals with a CDR score of 0. Furthermore, in this study, subjects with any serious neurological disease except for possible AD, any history of brain lesions or trauma, or psychoactive medication use (including antidepressants, neuroleptics, chronic anxiolytics, or sedative hypnotics) were excluded. In order to avoid population stratification effects which can lead to spurious genetic associations, we performed the principal component analysis (PCA). We assigned genotype-determined ancestry by comparing ADNI patients and populations form HapMap Phase 3 data and only individuals clustering with European HapMap samples were retained in our study.

### SNP selection and Genotyping

Seven AD associated SNPs were selected for analysis.They have been validated to associate with AD in ethnically distinct populations^7^,^8^,^9^,^10^,^11^,^21^,^28^,^67^: rs2279590, rs11136000, rs9331888, rs7012010, rs9331949, rs7982, rs1532278. *CLU* genotypes were extracted from the ADNI GWAS PLINK format data^68^. We performed the quality control (QC) procedures using PLINK software. The inclusion criteria were as follows: minimum call rates >90%, minimum minor allele frequencies (MAF) > 0.01, Hardy-Weinberg equilibrium test P > 0.001.

### PET measure-Aβ deposition

PET imaging data with amyloid tracer, florbetapir (AV-45), were obtained from UC Berkeley-AV45 analysis dataset on website (http://adni.loni.usc.edu/data-samples/access-data/). This institute used a native-space MRI scan for each subject which is segmented with Freesurfer (version 4.5.0) to define cortical grey matter regions of interest (ROI) (frontal, anterior/posterior cingulate, lateral parietal, lateral temporal) that make up a summary cortical ROI^69^,^70^. Notebly, the whole cerebellum was defined as reference region. Each florbetapir scan was applied to the corresponding MRI and mean florbetapir uptake within the cortical and reference region was calculated. Finally, SUVRs were created by averaging across the 4 cortical regions and dividing the cortical summary ROI by the whole cerebellum.

### CSF Protein

CSF samples were collected and transported to the ADNI Biomarker Core laboratory at the University of Pennsylvania Medical Center in dry ice. Preparation of aliquots (0.5 ml) from the collected samples was conducted after thawing (1 h) at room temperature and gentle mixing. The aliquots were stored in bar code–labeled polypropylene vials at −80° C environment. The CSF proteins, including Aβ1-42, Total-tau and Phosphorylated tau181p, were calculated using the multiplex xMAP Luminex platform (Luminex Corp, Austin, TX) with Innogenetics (INNO-BIA AlzBio3; Ghent, Belgium; for research use-only reagents) immunoassay kit–based reagents. Additional analysis details and quality control procedures are showed at site (http://adni.loni.ucla.edu).The measurements of CSF biomarker for this article were cross-sectional from the baseline evaluation. Finally, a total of 501 individuals with genetic and other information were included in CSF analysis from the ADNI sites.

### MRI structure

Our study used UCSF FreeSurfer datasets to conduct association test of *CLU* genotypes with brain structure. The cerebral image segmentation and analysis were performed with the FreeSurfer version 5.1 (http://surfer.nmr.mgh.harvard.edu/) based on the 2010 Desikan-Killany atlas^71^. We obtained data from motion correction and averaging of multiple volumetric T1 weighted images (when more than one is available), removal of non-brain tissue using a hybrid watershed/surface deformation procedure, automated Talairach transformation, segmentation of the subcortical white matter and deep gray matter volumetric structures (including hippocampus, amygdala, caudate, putamen, ventricles)^72^, intensity normalization, tessellation of the gray matter white matter boundary, automated topology correction, and surface deformation following intensity gradients to optimally place the gray/white as well as gray/cerebrospinal fluid borders at the location where the greatest shift in intensity defines the transition to the other tissue class. The technical details of these procedures are described in prior publications^73^.

### PET measure-Glucose metabolism

FDG analysis data were from UC Berkeley and Lawrence Berkeley National Laboratory on the website (http://adni.loni.usc.edu/data-samples/access-data/)^74^. In this laboratory, five regions (left and right angular gyrus, bilateral posterior cingulate, left and right temporal gyrus) were treated as metaROIs (regions of interest) to analysis. Firstly, we downloaded the PET data from LONI (http://loni.usc.edu/). Then these images were spatially normalized in SPM to the MNI PET template. The mean counts from the metaROIs for each subject’s FDG scans at each time point were extracted and the intensity values were computed with SPM subroutines. Finally, the mean of the top 50% of voxels within a hand-drawn pons/cerebellar vermis region which was hand-drawn on a T1 template in MNI space was extracted. In addition, each metaROI mean was normalized by dividing it by pons/vermis reference region mean^75^.

### Statistical Analysis

Differences in continuous variables were examined using one-way analysis of variance (ANOVA), and categorical data were tested using χ^2^ test. ADNI sample were stratified into three groups (CN, MCI and AD) to detect the effects of *CLU* genetic variations on neuroimaging phenotypes in the three clinical stages respectively. Moreover, we used a multiple linear regression model which considered age, gender, education, and *ApoE* ε4 status as covariates to estimate coefficients for testing possible correlation between various phenotypes and *CLU* genotypes. All statistical analyses were performed by R 3.12 and PLINK 8 (http://pngu.mgh.harvard.edu/wpurcell/plink/). To control multiple hypothesis testing, we used the false discovery rate (FDR) for correction^76^ and statistical significance was defined for FDR-corrected P < 0.05.

## Additional Information

**How to cite this article**: Tan, L. *et al*. Effect of CLU genetic variants on cerebrospinal fluid and neuroimaging markers in healthy, mild cognitive impairment and Alzheimer’s disease cohorts. *Sci. Rep*. **6**, 26027; doi: 10.1038/srep26027 (2016).

## Supplementary Material

Supplementary Information

## Figures and Tables

**Table 1:** The characteristics of included seven SNPs.

SNP	Position	Minor allele	MAF				H-W (p value)				Previous studied articles (PMID)
			All	AD	MCI	NC	All	AD	MCI	NC	
rs2279590	intron variant	T	0.379	0.365	0.375	0.39	0.221	0.638	0.534	0.444	[22015308], [20599866], [19734903], [21300948], [20697030]
rs11136000	intron variant	T	0.395	0.344	0.391	0.411	0.140	0.500	0.524	0.254	[21460841], [25189118], [25496871], [19734903] [20697030], [24806679], [24670887], [24117116], [23892938], [23650005], [23643458], [22722634], [22015308], [19734902]
rs9331888	intron variant, nc transcript variant, upstream variant	G	0.275	0.362	0.274	0.263	0.966	0.350	0.330	0.073	[22258514], [22122982], [20599866], [21892414]
rs7012010	nc transcript variant	C	0.306	0.271	0.318	0.292	0.945	0.932	0.381	0.437	[20697030], [19734902]
rs9331949	nc transcript variant, utr variant 3 prime	G	0.027	0.021	0.018	0.043	0.210	1.000	1.000	0.165	[23411014]
rs7982	nc transcript variant, synonymous codon	A	0.385	0.344	0.383	0.395	0.043	0.500	0.229	0.191	[20697030], [19734902]
rs1532278	intron variant	T	0.375	0.344	0.373	0.384	0.151	0.500	0.385	0.467	[21460841], [24806679]

Abbreviations: SNP, single nucleotidepolymorphism; MAF, minor allele frequency; AD, Alzheimer’s disease; MCI, mild cognitive impairment; NC, normal control.

**Table 2:** The significant associations of *CLU* loci with Aβ deposition in the whole group.

ROI	SNP	Baseline of the whole group			
		Beta	Sample	P	FDR-P
Frontal (SUVR)	rs11136000	−0.039	574	0.023	0.052
	rs1532278	−0.037	574	0.030	0.052
	rs2279590	−0.045	574	0.009	0.037
	rs7982	−0.044	574	0.011	0.037
Cingulate (SUVR)	rs1532278	−0.039	574	0.033	0.079
	rs2279590	−0.051	574	0.005	0.035
	rs7982	−0.044	574	0.018	0.063
Parietal (SUVR)	rs7982	−0.035	574	0.047	0.137
Temporal (SUVR)	rs2279590	−0.032	574	0.041	0.183
Summary (SUVR)	rs11136000	−0.032	574	0.009	0.030
	rs1532278	−0.029	574	0.023	0.039
	rs2279590	−0.031	574	0.013	0.030
	rs7982	−0.035	574	0.006	0.030
	rs9331888	0.021	572	0.018	0.042

ROI = regions of interest; SUVR = florbetapir standard uptake value ratios.

The blue color means the P value is still significant after FDR correction.
