# Supplementary material for: Effect of CLU genetic variants on cerebrospinal fluid and neuroimaging markers in healthy, mild cognitive impairment and Alzheimer’s disease cohorts
Source: Sci Rep. 2016 May 27;6:26027. doi: 10.1038/srep26027 (PMC4882617; doi:10.1038/srep26027)
Supplement: Supplementary Information [file srep26027-s1.doc]

**Effect of CLU genetic variants on cerebrospinal fluid and neuroimaging markers in healthy, mild cognitive impairment and Alzheimer's disease cohorts**

Lin Tan^1,#^, Hui-Fu Wang^2,#^, Meng-Shan Tan^3^, Chen-Chen Tan^3^, Xi-Chen Zhu^2^, Miao Dan^3^, Wan-Jiang Yu^4^, Teng Jiang^5^, Lan Tan^1,2,3^,*, Jin-Tai Yu^1,2,3,6^,*, Alzheimer’s Disease Neuroimaging Initiative

^1^ College of Medicine and Pharmaceutics, Ocean University of China, China

^2^ Department of Neurology, Qingdao Municipal Hospital, Nanjing Medical University, Qingdao, China

^3^ Department of Neurology, Qingdao Municipal Hospital, School of Medicine, Qingdao University, Qingdao, China

^4^ Department of Radiology, Qingdao Municipal Hospital, School of Medicine, Qingdao University, Qingdao, China

^5^ Department of Neurology, Nanjing First Hospital, Nanjing Medical University, Nanjing, China

^6^ Memory and Aging Center, Department of Neurology, University of California, San Francisco, CA, USA

# The first two authors contributed equally to this work.

* Address correspondence to Dr Lan Tan MD, PhD, Department of Neurology, Qingdao Municipal Hospital, School of Medicine, Qingdao University, No.5 Donghai Middle Road, Qingdao, Shandong Province 266071, China; or Jin-Tai Yu, MD, PhD, Department of Neurology, University of California, San Francisco, 675 Nelson Rising Lane, Suite 190, Box 1207, San Francisco, CA 94158, USA.

E-mail addresses: dr.tanlan@163.com (L. Tan); jintai.yu@ucsf.edu (J.T. Yu);

Tel: +86-532-8890-5659; Fax: +86-532-8890-5659.

**Table S1. The characteristics of the ADNI subjects at baseline**

| Characteristics | CN | | MCI | | AD | | P^*^ |
| --- | --- | --- | --- | --- | --- | --- | --- |
| Age (years) | 281 | 74.51±5.56 | 483 | 72.28±7.45 | 48 | 75.51±9.23 | - |
| Gender (male/female) | 281 | 136/145 | 483 | 282/201 | 48 | 30/18 | - |
| Education (years) | 281 | 16.41±2.66 | 483 | 15.98±2.82 | 48 | 15.73±2.62 | 0.08 |
| ApoE ε4 (0/1/2) | 281 | 204/70/7 | 483 | 262/180/41 | 48 | 14/25/9 | <0.01 |
| CDRSB (scores) | 207 | 6.54±0.55 | 406 | 6.32±0.64 | 47 | 5.3±0.72 | <0.01 |
| ADAS (scores) | 281 | 29.07±1.15 | 483 | 27.89±1.69 | 48 | 22.96±2.03 | <0.01 |
| MMSE (scores) | 281 | 9.06±4.23 | 480 | 15.3±6.65 | 48 | 29.8±8.44 | <0.01 |
| RAVLT total (scores) | 280 | 44.83±9.6 | 483 | 36.16±10.86 | 47 | 22.32±7.84 | <0.01 |
| FAQ (scores) | 281 | 0.17±0.66 | 481 | 2.85±3.99 | 48 | 12.6±7.14 | <0.01 |
| Hippocampus (mm^3^) | 257 | 7344±895 | 422 | 6996±1126 | 39 | 5757±948 | <0.01 |
| Middle Temporal (mm^3^) | 257 | 20298±2600 | 422 | 20186±2735 | 39 | 17776±3230 | <0.01 |
| Entorhinal (mm^3^) | 257 | 3803±650 | 422 | 3610±723 | 39 | 2919±705 | <0.01 |
| FDG | 207 | 6.55±0.55 | 406 | 6.32±0.64 | 47 | 5.3±0.72 | <0.01 |

Note: CN, cognitively normal; MCI, mild cognition impairment; AD, Alzheimer’s disease; CDRSB, Clinical Dementia Rating scale sum of boxes; ADAS, Alzheimer’s disease Assessment Scale; MMSE, Mini-Mental State Exam; RAVLT, Rey Auditory Verbal Learning Test; FAQ, Functional Activities Questionnaire; FDG, Cerebral Glucose Metabolism Rate measured with fluorodeoxyglucose-positron emission tomography(FDG-PET).

^*^P values for continuous variables are from one-way analysis of variance (ANOVA). P values for categorical data are from chi square test.

Data are given as mean ± standard deviation unless otherwise indicated

**Table S2. Association between *CLU* genetic variants and Aβ accumulation on AV45 at baseline**

| Region | SNP | BP | N | P | Pc |
| --- | --- | --- | --- | --- | --- |
| cingulate | rs2279590 | 27456253 | 574 | 0.005 | 0.035 |
| cingulate | rs7982 | 27462481 | 574 | 0.018 | 0.063 |
| cingulate | rs1532278 | 27466315 | 574 | 0.034 | 0.079 |
| cingulate | rs11136000 | 27464519 | 574 | 0.055 | 0.096 |
| cingulate | rs9331888 | 27468862 | 572 | 0.357 | 0.5 |
| cingulate | rs7012010 | 27448729 | 574 | 0.646 | 0.754 |
| cingulate | rs9331949 | 27454686 | 570 | 0.869 | 0.869 |
| frontal | rs2279590 | 27456253 | 574 | 0.009 | 0.037 |
| frontal | rs7982 | 27462481 | 574 | 0.011 | 0.037 |
| frontal | rs11136000 | 27464519 | 574 | 0.023 | 0.052 |
| frontal | rs1532278 | 27466315 | 574 | 0.03 | 0.052 |
| frontal | rs9331888 | 27468862 | 572 | 0.166 | 0.233 |
| frontal | rs7012010 | 27448729 | 574 | 0.521 | 0.608 |
| frontal | rs9331949 | 27454686 | 570 | 0.873 | 0.873 |
| parietal | rs7982 | 27462481 | 574 | 0.047 | 0.137 |
| parietal | rs2279590 | 27456253 | 574 | 0.05 | 0.137 |
| parietal | rs11136000 | 27464519 | 574 | 0.059 | 0.137 |
| parietal | rs1532278 | 27466315 | 574 | 0.099 | 0.174 |
| parietal | rs9331888 | 27468862 | 572 | 0.25 | 0.35 |
| parietal | rs9331949 | 27454686 | 570 | 0.893 | 0.986 |
| parietal | rs7012010 | 27448729 | 574 | 0.986 | 0.986 |
| summarysuvr_composite_refnorm | rs11136000 | 27464519 | 574 | 0.005 | 0.022 |
| summarysuvr_composite_refnorm | rs7982 | 27462481 | 574 | 0.006 | 0.022 |
| summarysuvr_composite_refnorm | rs9331888 | 27468862 | 572 | 0.018 | 0.042 |
| summarysuvr_composite_refnorm | rs1532278 | 27466315 | 574 | 0.034 | 0.059 |
| summarysuvr_composite_refnorm | rs2279590 | 27456253 | 574 | 0.043 | 0.061 |
| summarysuvr_composite_refnorm | rs7012010 | 27448729 | 574 | 0.724 | 0.734 |
| summarysuvr_composite_refnorm | rs9331949 | 27454686 | 570 | 0.734 | 0.734 |
| summarysuvr_wholecerebnorm | rs7982 | 27462481 | 574 | 0.006 | 0.03 |
| summarysuvr_wholecerebnorm | rs11136000 | 27464519 | 574 | 0.009 | 0.03 |
| summarysuvr_wholecerebnorm | rs2279590 | 27456253 | 574 | 0.013 | 0.03 |
| summarysuvr_wholecerebnorm | rs1532278 | 27466315 | 574 | 0.023 | 0.039 |
| summarysuvr_wholecerebnorm | rs9331888 | 27468862 | 572 | 0.121 | 0.169 |
| summarysuvr_wholecerebnorm | rs7012010 | 27448729 | 574 | 0.475 | 0.554 |
| summarysuvr_wholecerebnorm | rs9331949 | 27454686 | 570 | 0.679 | 0.679 |
| temporal | rs2279590 | 27456253 | 574 | 0.041 | 0.183 |
| temporal | rs7982 | 27462481 | 574 | 0.058 | 0.183 |
| temporal | rs11136000 | 27464519 | 574 | 0.079 | 0.183 |
| temporal | rs1532278 | 27466315 | 574 | 0.11 | 0.192 |
| temporal | rs9331888 | 27468862 | 572 | 0.242 | 0.338 |
| temporal | rs9331949 | 27454686 | 570 | 0.818 | 0.954 |
| temporal | rs7012010 | 27448729 | 574 | 0.96 | 0.96 |

NOTE:Pc value is the P value after false discovery rate (FDR) correction.

**Table S3. Association between *CLU* genetic variants and Aβ accumulation on AV45 at two-year followup study**

| Region | SNP | BP | N | P | Pc |
| --- | --- | --- | --- | --- | --- |
| cingulate | rs9331888 | 27468862 | 395 | 0.015 | 0.106 |
| cingulate | rs2279590 | 27456253 | 397 | 0.176 | 0.541 |
| cingulate | rs7012010 | 27448729 | 397 | 0.232 | 0.541 |
| cingulate | rs9331949 | 27454686 | 393 | 0.506 | 0.591 |
| cingulate | rs11136000 | 27464519 | 397 | 0.512 | 0.591 |
| cingulate | rs1532278 | 27466315 | 397 | 0.563 | 0.591 |
| cingulate | rs7982 | 27462481 | 397 | 0.591 | 0.591 |
| frontal | rs9331888 | 27468862 | 395 | 0.007 | 0.05 |
| frontal | rs2279590 | 27456253 | 397 | 0.189 | 0.581 |
| frontal | rs7012010 | 27448729 | 397 | 0.282 | 0.581 |
| frontal | rs11136000 | 27464519 | 397 | 0.52 | 0.581 |
| frontal | rs1532278 | 27466315 | 397 | 0.527 | 0.581 |
| frontal | rs7982 | 27462481 | 397 | 0.541 | 0.581 |
| frontal | rs9331949 | 27454686 | 393 | 0.581 | 0.581 |
| parietal | rs9331888 | 27468862 | 395 | 0.013 | 0.092 |
| parietal | rs2279590 | 27456253 | 397 | 0.226 | 0.601 |
| parietal | rs7012010 | 27448729 | 397 | 0.258 | 0.601 |
| parietal | rs9331949 | 27454686 | 393 | 0.413 | 0.664 |
| parietal | rs1532278 | 27466315 | 397 | 0.65 | 0.664 |
| parietal | rs7982 | 27462481 | 397 | 0.663 | 0.664 |
| parietal | rs11136000 | 27464519 | 397 | 0.664 | 0.664 |
| summarysuvr_composite_refnorm | rs7012010 | 27448729 | 397 | 0.402 | 0.849 |
| summarysuvr_composite_refnorm | rs9331949 | 27454686 | 393 | 0.492 | 0.849 |
| summarysuvr_composite_refnorm | rs11136000 | 27464519 | 397 | 0.736 | 0.849 |
| summarysuvr_composite_refnorm | rs7982 | 27462481 | 397 | 0.756 | 0.849 |
| summarysuvr_composite_refnorm | rs2279590 | 27456253 | 397 | 0.769 | 0.849 |
| summarysuvr_composite_refnorm | rs9331888 | 27468862 | 395 | 0.842 | 0.849 |
| summarysuvr_composite_refnorm | rs1532278 | 27466315 | 397 | 0.849 | 0.849 |
| summarysuvr_wholecerebnorm | rs9331888 | 27468862 | 395 | 0.074 | 0.518 |
| summarysuvr_wholecerebnorm | rs2279590 | 27456253 | 397 | 0.409 | 0.916 |
| summarysuvr_wholecerebnorm | rs9331949 | 27454686 | 393 | 0.425 | 0.916 |
| summarysuvr_wholecerebnorm | rs7012010 | 27448729 | 397 | 0.571 | 0.916 |
| summarysuvr_wholecerebnorm | rs11136000 | 27464519 | 397 | 0.87 | 0.916 |
| summarysuvr_wholecerebnorm | rs1532278 | 27466315 | 397 | 0.915 | 0.916 |
| summarysuvr_wholecerebnorm | rs7982 | 27462481 | 397 | 0.916 | 0.916 |
| temporal | rs9331888 | 27468862 | 395 | 0.004 | 0.031 |
| temporal | rs7012010 | 27448729 | 397 | 0.274 | 0.657 |
| temporal | rs2279590 | 27456253 | 397 | 0.296 | 0.657 |
| temporal | rs9331949 | 27454686 | 393 | 0.588 | 0.657 |
| temporal | rs11136000 | 27464519 | 397 | 0.602 | 0.657 |
| temporal | rs7982 | 27462481 | 397 | 0.617 | 0.657 |
| temporal | rs1532278 | 27466315 | 397 | 0.657 | 0.657 |

NOTE:Pc value is the P value after false discovery rate (FDR) correction.

**Table S4. Association between *CLU*** genetic variants and cortex volume on MRI at baseline

| Region | SNP | BP | N | P | Pc |
| --- | --- | --- | --- | --- | --- |
| Volume of LeftEntorhinal | rs9331888 | 27468862 | 534 | 0.153 | 0.971 |
| Volume of LeftEntorhinal | rs1532278 | 27466315 | 536 | 0.548 | 0.971 |
| Volume of LeftEntorhinal | rs7982 | 27462481 | 536 | 0.733 | 0.971 |
| Volume of LeftEntorhinal | rs2279590 | 27456253 | 536 | 0.736 | 0.971 |
| Volume of LeftEntorhinal | rs7012010 | 27448729 | 536 | 0.939 | 0.971 |
| Volume of LeftEntorhinal | rs9331949 | 27454686 | 533 | 0.958 | 0.971 |
| Volume of LeftEntorhinal | rs11136000 | 27464519 | 536 | 0.971 | 0.971 |
| Volume of LeftMiddleTemporal | rs9331888 | 27468862 | 534 | 0.075 | 0.524 |
| Volume of LeftMiddleTemporal | rs7012010 | 27448729 | 536 | 0.303 | 0.682 |
| Volume of LeftMiddleTemporal | rs11136000 | 27464519 | 536 | 0.395 | 0.682 |
| Volume of LeftMiddleTemporal | rs2279590 | 27456253 | 536 | 0.424 | 0.682 |
| Volume of LeftMiddleTemporal | rs7982 | 27462481 | 536 | 0.528 | 0.682 |
| Volume of LeftMiddleTemporal | rs1532278 | 27466315 | 536 | 0.585 | 0.682 |
| Volume of LeftMiddleTemporal | rs9331949 | 27454686 | 533 | 0.89 | 0.89 |
| Volume of LeftParahippocampal | rs9331888 | 27468862 | 534 | 0.32 | 0.87 |
| Volume of LeftParahippocampal | rs2279590 | 27456253 | 536 | 0.511 | 0.87 |
| Volume of LeftParahippocampal | rs11136000 | 27464519 | 536 | 0.538 | 0.87 |
| Volume of LeftParahippocampal | rs7982 | 27462481 | 536 | 0.578 | 0.87 |
| Volume of LeftParahippocampal | rs1532278 | 27466315 | 536 | 0.657 | 0.87 |
| Volume of LeftParahippocampal | rs9331949 | 27454686 | 533 | 0.746 | 0.87 |
| Volume of LeftParahippocampal | rs7012010 | 27448729 | 536 | 0.959 | 0.959 |
| Volume of LeftPosteriorCingulate | rs9331888 | 27468862 | 534 | 0.121 | 0.621 |
| Volume of LeftPosteriorCingulate | rs11136000 | 27464519 | 536 | 0.311 | 0.621 |
| Volume of LeftPosteriorCingulate | rs7012010 | 27448729 | 536 | 0.343 | 0.621 |
| Volume of LeftPosteriorCingulate | rs7982 | 27462481 | 536 | 0.496 | 0.621 |
| Volume of LeftPosteriorCingulate | rs1532278 | 27466315 | 536 | 0.516 | 0.621 |
| Volume of LeftPosteriorCingulate | rs9331949 | 27454686 | 533 | 0.602 | 0.621 |
| Volume of LeftPosteriorCingulate | rs2279590 | 27456253 | 536 | 0.621 | 0.621 |
| Volume of LeftPrecuneus | rs2279590 | 27456253 | 536 | 0.339 | 0.909 |
| Volume of LeftPrecuneus | rs7012010 | 27448729 | 536 | 0.481 | 0.909 |
| Volume of LeftPrecuneus | rs9331949 | 27454686 | 533 | 0.541 | 0.909 |
| Volume of LeftPrecuneus | rs11136000 | 27464519 | 536 | 0.618 | 0.909 |
| Volume of LeftPrecuneus | rs7982 | 27462481 | 536 | 0.662 | 0.909 |
| Volume of LeftPrecuneus | rs1532278 | 27466315 | 536 | 0.779 | 0.909 |
| Volume of LeftPrecuneus | rs9331888 | 27468862 | 534 | 0.985 | 0.985 |
| Volume of RightEntorhinal | rs9331888 | 27468862 | 534 | 0.088 | 0.613 |
| Volume of RightEntorhinal | rs2279590 | 27456253 | 536 | 0.387 | 0.647 |
| Volume of RightEntorhinal | rs7982 | 27462481 | 536 | 0.405 | 0.647 |
| Volume of RightEntorhinal | rs11136000 | 27464519 | 536 | 0.571 | 0.647 |
| Volume of RightEntorhinal | rs9331949 | 27454686 | 533 | 0.631 | 0.647 |
| Volume of RightEntorhinal | rs7012010 | 27448729 | 536 | 0.633 | 0.647 |
| Volume of RightEntorhinal | rs1532278 | 27466315 | 536 | 0.647 | 0.647 |
| Volume of RightMiddleTemporal | rs2279590 | 27456253 | 536 | 0.161 | 0.478 |
| Volume of RightMiddleTemporal | rs9331888 | 27468862 | 534 | 0.298 | 0.478 |
| Volume of RightMiddleTemporal | rs1532278 | 27466315 | 536 | 0.386 | 0.478 |
| Volume of RightMiddleTemporal | rs7012010 | 27448729 | 536 | 0.397 | 0.478 |
| Volume of RightMiddleTemporal | rs7982 | 27462481 | 536 | 0.408 | 0.478 |
| Volume of RightMiddleTemporal | rs11136000 | 27464519 | 536 | 0.41 | 0.478 |
| Volume of RightMiddleTemporal | rs9331949 | 27454686 | 533 | 0.935 | 0.935 |
| Volume of RightParahippocampal | rs9331888 | 27468862 | 534 | 0.134 | 0.416 |
| Volume of RightParahippocampal | rs7012010 | 27448729 | 536 | 0.148 | 0.416 |
| Volume of RightParahippocampal | rs2279590 | 27456253 | 536 | 0.27 | 0.416 |
| Volume of RightParahippocampal | rs11136000 | 27464519 | 536 | 0.274 | 0.416 |
| Volume of RightParahippocampal | rs7982 | 27462481 | 536 | 0.299 | 0.416 |
| Volume of RightParahippocampal | rs1532278 | 27466315 | 536 | 0.356 | 0.416 |
| Volume of RightParahippocampal | rs9331949 | 27454686 | 533 | 0.74 | 0.74 |
| Volume of RightPosteriorCingulate | rs2279590 | 27456253 | 536 | 0.363 | 0.882 |
| Volume of RightPosteriorCingulate | rs9331888 | 27468862 | 534 | 0.406 | 0.882 |
| Volume of RightPosteriorCingulate | rs11136000 | 27464519 | 536 | 0.58 | 0.882 |
| Volume of RightPosteriorCingulate | rs7982 | 27462481 | 536 | 0.588 | 0.882 |
| Volume of RightPosteriorCingulate | rs9331949 | 27454686 | 533 | 0.861 | 0.882 |
| Volume of RightPosteriorCingulate | rs7012010 | 27448729 | 536 | 0.879 | 0.882 |
| Volume of RightPosteriorCingulate | rs1532278 | 27466315 | 536 | 0.882 | 0.882 |
| Volume of RightPrecuneus | rs2279590 | 27456253 | 536 | 0.264 | 0.993 |
| Volume of RightPrecuneus | rs9331949 | 27454686 | 533 | 0.546 | 0.993 |
| Volume of RightPrecuneus | rs1532278 | 27466315 | 536 | 0.552 | 0.993 |
| Volume of RightPrecuneus | rs9331888 | 27468862 | 534 | 0.586 | 0.993 |
| Volume of RightPrecuneus | rs7012010 | 27448729 | 536 | 0.808 | 0.993 |
| Volume of RightPrecuneus | rs11136000 | 27464519 | 536 | 0.869 | 0.993 |
| Volume of RightPrecuneus | rs7982 | 27462481 | 536 | 0.993 | 0.993 |

NOTE:Pc value is the P value after false discovery rate (FDR) correction.

**Table S5. Association between *CLU* genetic variants and cortex volume on MRI at two-year follow-up study**

| Region | SNP | BP | N | P | Pc |
| --- | --- | --- | --- | --- | --- |
| Volume of LeftEntorhinal | rs9331949 | 27454686 | 235 | 0.075 | 0.524 |
| Volume of LeftEntorhinal | rs11136000 | 27464519 | 238 | 0.615 | 0.955 |
| Volume of LeftEntorhinal | rs7012010 | 27448729 | 238 | 0.759 | 0.955 |
| Volume of LeftEntorhinal | rs7982 | 27462481 | 238 | 0.786 | 0.955 |
| Volume of LeftEntorhinal | rs1532278 | 27466315 | 238 | 0.887 | 0.955 |
| Volume of LeftEntorhinal | rs2279590 | 27456253 | 238 | 0.919 | 0.955 |
| Volume of LeftEntorhinal | rs9331888 | 27468862 | 237 | 0.955 | 0.955 |
| Volume of LeftMiddleTemporal | rs9331949 | 27454686 | 235 | 0.274 | 0.947 |
| Volume of LeftMiddleTemporal | rs2279590 | 27456253 | 238 | 0.525 | 0.947 |
| Volume of LeftMiddleTemporal | rs11136000 | 27464519 | 238 | 0.6 | 0.947 |
| Volume of LeftMiddleTemporal | rs1532278 | 27466315 | 238 | 0.618 | 0.947 |
| Volume of LeftMiddleTemporal | rs7982 | 27462481 | 238 | 0.742 | 0.947 |
| Volume of LeftMiddleTemporal | rs7012010 | 27448729 | 238 | 0.812 | 0.947 |
| Volume of LeftMiddleTemporal | rs9331888 | 27468862 | 237 | 0.967 | 0.967 |
| Volume of LeftParahippocampal | rs7012010 | 27448729 | 237 | 0.065 | 0.338 |
| Volume of LeftParahippocampal | rs9331949 | 27454686 | 234 | 0.201 | 0.338 |
| Volume of LeftParahippocampal | rs7982 | 27462481 | 237 | 0.255 | 0.338 |
| Volume of LeftParahippocampal | rs11136000 | 27464519 | 237 | 0.272 | 0.338 |
| Volume of LeftParahippocampal | rs1532278 | 27466315 | 237 | 0.273 | 0.338 |
| Volume of LeftParahippocampal | rs9331888 | 27468862 | 236 | 0.289 | 0.338 |
| Volume of LeftParahippocampal | rs2279590 | 27456253 | 237 | 0.431 | 0.431 |
| Volume of LeftPosteriorCingulate | rs7012010 | 27448729 | 238 | 0.099 | 0.695 |
| Volume of LeftPosteriorCingulate | rs9331888 | 27468862 | 237 | 0.495 | 0.917 |
| Volume of LeftPosteriorCingulate | rs11136000 | 27464519 | 238 | 0.75 | 0.917 |
| Volume of LeftPosteriorCingulate | rs1532278 | 27466315 | 238 | 0.754 | 0.917 |
| Volume of LeftPosteriorCingulate | rs9331949 | 27454686 | 235 | 0.777 | 0.917 |
| Volume of LeftPosteriorCingulate | rs7982 | 27462481 | 238 | 0.81 | 0.917 |
| Volume of LeftPosteriorCingulate | rs2279590 | 27456253 | 238 | 0.917 | 0.917 |
| Volume of LeftPrecuneus | rs9331888 | 27468862 | 237 | 0.451 | 0.964 |
| Volume of LeftPrecuneus | rs7012010 | 27448729 | 238 | 0.47 | 0.964 |
| Volume of LeftPrecuneus | rs7982 | 27462481 | 238 | 0.651 | 0.964 |
| Volume of LeftPrecuneus | rs1532278 | 27466315 | 238 | 0.741 | 0.964 |
| Volume of LeftPrecuneus | rs2279590 | 27456253 | 238 | 0.795 | 0.964 |
| Volume of LeftPrecuneus | rs9331949 | 27454686 | 235 | 0.869 | 0.964 |
| Volume of LeftPrecuneus | rs11136000 | 27464519 | 238 | 0.964 | 0.964 |
| Volume of RightEntorhinal | rs9331888 | 27468862 | 237 | 0.295 | 0.732 |
| Volume of RightEntorhinal | rs1532278 | 27466315 | 238 | 0.318 | 0.732 |
| Volume of RightEntorhinal | rs7982 | 27462481 | 238 | 0.397 | 0.732 |
| Volume of RightEntorhinal | rs11136000 | 27464519 | 238 | 0.418 | 0.732 |
| Volume of RightEntorhinal | rs2279590 | 27456253 | 238 | 0.668 | 0.906 |
| Volume of RightEntorhinal | rs7012010 | 27448729 | 238 | 0.854 | 0.906 |
| Volume of RightEntorhinal | rs9331949 | 27454686 | 235 | 0.906 | 0.906 |
| Volume of RightMiddleTemporal | rs7982 | 27462481 | 238 | 0.387 | 0.894 |
| Volume of RightMiddleTemporal | rs11136000 | 27464519 | 238 | 0.515 | 0.894 |
| Volume of RightMiddleTemporal | rs2279590 | 27456253 | 238 | 0.565 | 0.894 |
| Volume of RightMiddleTemporal | rs7012010 | 27448729 | 238 | 0.575 | 0.894 |
| Volume of RightMiddleTemporal | rs1532278 | 27466315 | 238 | 0.662 | 0.894 |
| Volume of RightMiddleTemporal | rs9331949 | 27454686 | 235 | 0.766 | 0.894 |
| Volume of RightMiddleTemporal | rs9331888 | 27468862 | 237 | 0.997 | 0.997 |
| Volume of RightParahippocampal | rs9331949 | 27454686 | 235 | 0.6 | 0.826 |
| Volume of RightParahippocampal | rs11136000 | 27464519 | 238 | 0.615 | 0.826 |
| Volume of RightParahippocampal | rs1532278 | 27466315 | 238 | 0.71 | 0.826 |
| Volume of RightParahippocampal | rs9331888 | 27468862 | 237 | 0.716 | 0.826 |
| Volume of RightParahippocampal | rs7982 | 27462481 | 238 | 0.783 | 0.826 |
| Volume of RightParahippocampal | rs2279590 | 27456253 | 238 | 0.819 | 0.826 |
| Volume of RightParahippocampal | rs7012010 | 27448729 | 238 | 0.826 | 0.826 |
| Volume of RightPosteriorCingulate | rs1532278 | 27466315 | 238 | 0.369 | 0.809 |
| Volume of RightPosteriorCingulate | rs9331949 | 27454686 | 235 | 0.528 | 0.809 |
| Volume of RightPosteriorCingulate | rs2279590 | 27456253 | 238 | 0.54 | 0.809 |
| Volume of RightPosteriorCingulate | rs11136000 | 27464519 | 238 | 0.613 | 0.809 |
| Volume of RightPosteriorCingulate | rs7982 | 27462481 | 238 | 0.74 | 0.809 |
| Volume of RightPosteriorCingulate | rs7012010 | 27448729 | 238 | 0.761 | 0.809 |
| Volume of RightPosteriorCingulate | rs9331888 | 27468862 | 237 | 0.809 | 0.809 |
| Volume of RightPrecuneus | rs9331888 | 27468862 | 237 | 0.304 | 0.931 |
| Volume of RightPrecuneus | rs9331949 | 27454686 | 235 | 0.453 | 0.931 |
| Volume of RightPrecuneus | rs7982 | 27462481 | 238 | 0.692 | 0.931 |
| Volume of RightPrecuneus | rs7012010 | 27448729 | 238 | 0.857 | 0.931 |
| Volume of RightPrecuneus | rs2279590 | 27456253 | 238 | 0.869 | 0.931 |
| Volume of RightPrecuneus | rs1532278 | 27466315 | 238 | 0.906 | 0.931 |
| Volume of RightPrecuneus | rs11136000 | 27464519 | 238 | 0.931 | 0.931 |

NOTE:Pc value is the P value after false discovery rate (FDR) correction.

**Table S6. Association between *CLU* genetic variants and Hippocampus volume on MRI at baseline**

| Region | SNP | BP | N | P | Pc |
| --- | --- | --- | --- | --- | --- |
| Volume of Left Hippocampus | rs11136000 | 27464519 | 536 | 0.238 | 0.672 |
| Volume of Left Hippocampus | rs1532278 | 27466315 | 536 | 0.384 | 0.672 |
| Volume of Left Hippocampus | rs2279590 | 27456253 | 536 | 0.741 | 0.741 |
| Volume of Left Hippocampus | rs7012010 | 27448729 | 536 | 0.53 | 0.741 |
| Volume of Left Hippocampus | rs7982 | 27462481 | 536 | 0.307 | 0.672 |
| Volume of Left Hippocampus | rs9331888 | 27468862 | 534 | 0.002 | 0.014 |
| Volume of Left Hippocampus | rs9331949 | 27454686 | 533 | 0.655 | 0.741 |
| Volume of Right Hippocampus | rs11136000 | 27464519 | 536 | 0.159 | 0.479 |
| Volume of Right Hippocampus | rs1532278 | 27466315 | 536 | 0.274 | 0.479 |
| Volume of Right Hippocampus | rs2279590 | 27456253 | 536 | 0.462 | 0.646 |
| Volume of Right Hippocampus | rs7012010 | 27448729 | 536 | 0.725 | 0.725 |
| Volume of Right Hippocampus | rs7982 | 27462481 | 536 | 0.237 | 0.479 |
| Volume of Right Hippocampus | rs9331888 | 27468862 | 534 | 0.06 | 0.422 |
| Volume of Right Hippocampus | rs9331949 | 27454686 | 533 | 0.616 | 0.718 |

NOTE: Pc value is the P value after false discovery rate (FDR) correction.

**Table S7. Association between *CLU* genetic variants and Hippocampus volume on MRI at two-year followup study**

| Region | SNP | BP | N | P | Pc |
| --- | --- | --- | --- | --- | --- |
| Volume of Left Hippocampus | rs11136000 | 27464519 | 238 | 0.898 | 0.943 |
| Volume of Left Hippocampus | rs1532278 | 27466315 | 238 | 0.706 | 0.943 |
| Volume of Left Hippocampus | rs2279590 | 27456253 | 238 | 0.943 | 0.943 |
| Volume of Left Hippocampus | rs7012010 | 27448729 | 238 | 0.416 | 0.943 |
| Volume of Left Hippocampus | rs7982 | 27462481 | 238 | 0.936 | 0.943 |
| Volume of Left Hippocampus | rs9331888 | 27468862 | 237 | 0.261 | 0.943 |
| Volume of Left Hippocampus | rs9331949 | 27454686 | 235 | 0.654 | 0.943 |
| Volume of Right Hippocampus | rs11136000 | 27464519 | 238 | 0.426 | 0.903 |
| Volume of Right Hippocampus | rs1532278 | 27466315 | 238 | 0.9 | 0.903 |
| Volume of Right Hippocampus | rs2279590 | 27456253 | 238 | 0.675 | 0.903 |
| Volume of Right Hippocampus | rs7012010 | 27448729 | 238 | 0.903 | 0.903 |
| Volume of Right Hippocampus | rs7982 | 27462481 | 238 | 0.547 | 0.903 |
| Volume of Right Hippocampus | rs9331888 | 27468862 | 237 | 0.727 | 0.903 |
| Volume of Right Hippocampus | rs9331949 | 27454686 | 235 | 0.705 | 0.903 |

NOTE: Pc value is the P value after false discovery rate (FDR) correction.

**Table S8. Association between *CLU* genetic variants and Hippocampal Subfields Volume of CA1 on MRI at baseline**

| Region | SNP | BP | N | P | Pc |
| --- | --- | --- | --- | --- | --- |
| Hippocampal Subfields Volume of LeftCA1 | rs11136000 | 27464519 | 536 | 0.497 | 0.601 |
| Hippocampal Subfields Volume of LeftCA1 | rs1532278 | 27466315 | 536 | 0.601 | 0.601 |
| Hippocampal Subfields Volume of LeftCA1 | rs2279590 | 27456253 | 536 | 0.562 | 0.601 |
| Hippocampal Subfields Volume of LeftCA1 | rs7012010 | 27448729 | 536 | 0.565 | 0.601 |
| Hippocampal Subfields Volume of LeftCA1 | rs7982 | 27462481 | 536 | 0.551 | 0.601 |
| Hippocampal Subfields Volume of LeftCA1 | rs9331888 | 27468862 | 534 | 0.072 | 0.507 |
| Hippocampal Subfields Volume of LeftCA1 | rs9331949 | 27454686 | 533 | 0.502 | 0.601 |
| Hippocampal Subfields Volume of RightCA1 | rs11136000 | 27464519 | 536 | 0.154 | 0.34 |
| Hippocampal Subfields Volume of RightCA1 | rs1532278 | 27466315 | 536 | 0.291 | 0.34 |
| Hippocampal Subfields Volume of RightCA1 | rs2279590 | 27456253 | 536 | 0.255 | 0.34 |
| Hippocampal Subfields Volume of RightCA1 | rs7012010 | 27448729 | 536 | 0.259 | 0.34 |
| Hippocampal Subfields Volume of RightCA1 | rs7982 | 27462481 | 536 | 0.196 | 0.34 |
| Hippocampal Subfields Volume of RightCA1 | rs9331888 | 27468862 | 534 | 0.277 | 0.34 |
| Hippocampal Subfields Volume of RightCA1 | rs9331949 | 27454686 | 533 | 0.441 | 0.441 |

NOTE: Pc value is the P value after false discovery rate (FDR) correction.

**Table S9. Association between *CLU*** genetic variants and Hippocampal Subfields Volume of CA1 on MRI at two-year followup study

| Region | SNP | BP | N | P | Pc |
| --- | --- | --- | --- | --- | --- |
| Hippocampal Subfields Volume of Left CA1 | rs11136000 | 27464519 | 237 | 0.916 | 0.946 |
| Hippocampal Subfields Volume of Left CA1 | rs1532278 | 27466315 | 237 | 0.806 | 0.946 |
| Hippocampal Subfields Volume of Left CA1 | rs2279590 | 27456253 | 237 | 0.644 | 0.946 |
| Hippocampal Subfields Volume of Left CA1 | rs7012010 | 27448729 | 237 | 0.638 | 0.946 |
| Hippocampal Subfields Volume of Left CA1 | rs7982 | 27462481 | 237 | 0.946 | 0.946 |
| Hippocampal Subfields Volume of Left CA1 | rs9331888 | 27468862 | 236 | 0.937 | 0.946 |
| Hippocampal Subfields Volume of Left CA1 | rs9331949 | 27454686 | 234 | 0.699 | 0.946 |
| Hippocampal Subfields Volume of Right CA1 | rs11136000 | 27464519 | 237 | 0.323 | 0.465 |
| Hippocampal Subfields Volume of Right CA1 | rs1532278 | 27466315 | 237 | 0.255 | 0.465 |
| Hippocampal Subfields Volume of Right CA1 | rs2279590 | 27456253 | 237 | 0.332 | 0.465 |
| Hippocampal Subfields Volume of Right CA1 | rs7012010 | 27448729 | 237 | 0.496 | 0.578 |
| Hippocampal Subfields Volume of Right CA1 | rs7982 | 27462481 | 237 | 0.308 | 0.465 |
| Hippocampal Subfields Volume of Right CA1 | rs9331888 | 27468862 | 236 | 0.13 | 0.465 |
| Hippocampal Subfields Volume of Right CA1 | rs9331949 | 27454686 | 234 | 0.671 | 0.671 |

NOTE: Pc value is the P value after false discovery rate (FDR) correction.

**Table S10. Association between *CLU* genetic variants and cortex thickness on MRI at baseline**

| Region | SNP | BP | N | P | Pc |
| --- | --- | --- | --- | --- | --- |
| Cortical Thickness Average of Right Parahippocampal | rs11136000 | 27464519 | 536 | 0.718 | 0.859 |
| Cortical Thickness Average of Right Parahippocampal | rs1532278 | 27466315 | 536 | 0.781 | 0.859 |
| Cortical Thickness Average of Right Parahippocampal | rs2279590 | 27456253 | 536 | 0.825 | 0.859 |
| Cortical Thickness Average of Right Parahippocampal | rs7012010 | 27448729 | 536 | 0.859 | 0.859 |
| Cortical Thickness Average of Right Parahippocampal | rs7982 | 27462481 | 536 | 0.729 | 0.859 |
| Cortical Thickness Average of Right Parahippocampal | rs9331888 | 27468862 | 534 | 0.236 | 0.827 |
| Cortical Thickness Average of Right Parahippocampal | rs9331949 | 27454686 | 533 | 0.116 | 0.81 |
| Cortical Thickness Average of Right Posterior Cingulate | rs11136000 | 27464519 | 536 | 0.388 | 0.67 |
| Cortical Thickness Average of Right Posterior Cingulate | rs1532278 | 27466315 | 536 | 0.816 | 0.922 |
| Cortical Thickness Average of Right Posterior Cingulate | rs2279590 | 27456253 | 536 | 0.922 | 0.922 |
| Cortical Thickness Average of Right Posterior Cingulate | rs7012010 | 27448729 | 536 | 0.323 | 0.67 |
| Cortical Thickness Average of Right Posterior Cingulate | rs7982 | 27462481 | 536 | 0.479 | 0.67 |
| Cortical Thickness Average of Right Posterior Cingulate | rs9331888 | 27468862 | 534 | 0.231 | 0.67 |
| Cortical Thickness Average of Right Posterior Cingulate | rs9331949 | 27454686 | 533 | 0.238 | 0.67 |
| Cortical Thickness Average of Right Precuneus | rs11136000 | 27464519 | 536 | 0.137 | 0.265 |
| Cortical Thickness Average of Right Precuneus | rs1532278 | 27466315 | 536 | 0.038 | 0.187 |
| Cortical Thickness Average of Right Precuneus | rs2279590 | 27456253 | 536 | 0.053 | 0.187 |
| Cortical Thickness Average of Right Precuneus | rs7012010 | 27448729 | 536 | 0.507 | 0.507 |
| Cortical Thickness Average of Right Precuneus | rs7982 | 27462481 | 536 | 0.151 | 0.265 |
| Cortical Thickness Average of Right Precuneus | rs9331888 | 27468862 | 534 | 0.295 | 0.345 |
| Cortical Thickness Average of Right Precuneus | rs9331949 | 27454686 | 533 | 0.239 | 0.335 |
| Cortical Thickness Average of Left Entorhinal | rs11136000 | 27464519 | 536 | 0.712 | 0.86 |
| Cortical Thickness Average of Left Entorhinal | rs1532278 | 27466315 | 536 | 0.86 | 0.86 |
| Cortical Thickness Average of Left Entorhinal | rs2279590 | 27456253 | 536 | 0.846 | 0.86 |
| Cortical Thickness Average of Left Entorhinal | rs7012010 | 27448729 | 536 | 0.577 | 0.86 |
| Cortical Thickness Average of Left Entorhinal | rs7982 | 27462481 | 536 | 0.677 | 0.86 |
| Cortical Thickness Average of Left Entorhinal | rs9331888 | 27468862 | 534 | 0.016 | 0.113 |
| Cortical Thickness Average of Left Entorhinal | rs9331949 | 27454686 | 533 | 0.198 | 0.695 |
| Cortical Thickness Average of Left Middle Temporal | rs11136000 | 27464519 | 536 | 0.692 | 0.828 |
| Cortical Thickness Average of Left Middle Temporal | rs1532278 | 27466315 | 536 | 0.69 | 0.828 |
| Cortical Thickness Average of Left Middle Temporal | rs2279590 | 27456253 | 536 | 0.828 | 0.828 |
| Cortical Thickness Average of Left Middle Temporal | rs7012010 | 27448729 | 536 | 0.587 | 0.828 |
| Cortical Thickness Average of Left Middle Temporal | rs7982 | 27462481 | 536 | 0.722 | 0.828 |
| Cortical Thickness Average of Left Middle Temporal | rs9331888 | 27468862 | 534 | 0.606 | 0.828 |
| Cortical Thickness Average of Left Middle Temporal | rs9331949 | 27454686 | 533 | 0.26 | 0.828 |
| Cortical Thickness Average of Left Parahippocampal | rs11136000 | 27464519 | 536 | 0.139 | 0.506 |
| Cortical Thickness Average of Left Parahippocampal | rs1532278 | 27466315 | 536 | 0.458 | 0.79 |
| Cortical Thickness Average of Left Parahippocampal | rs2279590 | 27456253 | 536 | 0.53 | 0.79 |
| Cortical Thickness Average of Left Parahippocampal | rs7012010 | 27448729 | 536 | 0.677 | 0.79 |
| Cortical Thickness Average of Left Parahippocampal | rs7982 | 27462481 | 536 | 0.144 | 0.506 |
| Cortical Thickness Average of Left Parahippocampal | rs9331888 | 27468862 | 534 | 0.889 | 0.889 |
| Cortical Thickness Average of Left Parahippocampal | rs9331949 | 27454686 | 533 | 0.577 | 0.79 |
| Cortical Thickness Average of Left Posterior Cingulate | rs11136000 | 27464519 | 536 | 0.837 | 0.984 |
| Cortical Thickness Average of Left Posterior Cingulate | rs1532278 | 27466315 | 536 | 0.424 | 0.921 |
| Cortical Thickness Average of Left Posterior Cingulate | rs2279590 | 27456253 | 536 | 0.51 | 0.921 |
| Cortical Thickness Average of Left Posterior Cingulate | rs7012010 | 27448729 | 536 | 0.984 | 0.984 |
| Cortical Thickness Average of Left Posterior Cingulate | rs7982 | 27462481 | 536 | 0.927 | 0.984 |
| Cortical Thickness Average of Left Posterior Cingulate | rs9331888 | 27468862 | 534 | 0.214 | 0.921 |
| Cortical Thickness Average of Left Posterior Cingulate | rs9331949 | 27454686 | 533 | 0.526 | 0.921 |
| Cortical Thickness Average of Left Precuneus | rs11136000 | 27464519 | 536 | 0.349 | 0.489 |
| Cortical Thickness Average of Left Precuneus | rs1532278 | 27466315 | 536 | 0.065 | 0.456 |
| Cortical Thickness Average of Left Precuneus | rs2279590 | 27456253 | 536 | 0.152 | 0.489 |
| Cortical Thickness Average of Left Precuneus | rs7012010 | 27448729 | 536 | 0.609 | 0.71 |
| Cortical Thickness Average of Left Precuneus | rs7982 | 27462481 | 536 | 0.284 | 0.489 |
| Cortical Thickness Average of Left Precuneus | rs9331888 | 27468862 | 534 | 0.853 | 0.853 |
| Cortical Thickness Average of Left Precuneus | rs9331949 | 27454686 | 533 | 0.294 | 0.489 |
| Cortical Thickness Average of Right Entorhinal | rs11136000 | 27464519 | 536 | 0.89 | 0.89 |
| Cortical Thickness Average of Right Entorhinal | rs1532278 | 27466315 | 536 | 0.629 | 0.89 |
| Cortical Thickness Average of Right Entorhinal | rs2279590 | 27456253 | 536 | 0.65 | 0.89 |
| Cortical Thickness Average of Right Entorhinal | rs7012010 | 27448729 | 536 | 0.814 | 0.89 |
| Cortical Thickness Average of Right Entorhinal | rs7982 | 27462481 | 536 | 0.859 | 0.89 |
| Cortical Thickness Average of Right Entorhinal | rs9331888 | 27468862 | 534 | 0.077 | 0.542 |
| Cortical Thickness Average of Right Entorhinal | rs9331949 | 27454686 | 533 | 0.408 | 0.89 |
| Cortical Thickness Average of Right Middle Temporal | rs11136000 | 27464519 | 536 | 0.999 | 0.999 |
| Cortical Thickness Average of Right Middle Temporal | rs1532278 | 27466315 | 536 | 0.63 | 0.999 |
| Cortical Thickness Average of Right Middle Temporal | rs2279590 | 27456253 | 536 | 0.55 | 0.999 |
| Cortical Thickness Average of Right Middle Temporal | rs7012010 | 27448729 | 536 | 0.889 | 0.999 |
| Cortical Thickness Average of Right Middle Temporal | rs7982 | 27462481 | 536 | 0.997 | 0.999 |
| Cortical Thickness Average of Right Middle Temporal | rs9331888 | 27468862 | 534 | 0.59 | 0.999 |
| Cortical Thickness Average of Right Middle Temporal | rs9331949 | 27454686 | 533 | 0.494 | 0.999 |

NOTE: Pc value is the P value after false discovery rate (FDR) correction.

**Table S11. Association between *CLU* genetic variants and cortex thickness on MRI at two-year followup study**

| Region | SNP | BP | N | P | Pc |
| --- | --- | --- | --- | --- | --- |
| Cortical Thickness Average of Right Parahippocampal | rs11136000 | 27464519 | 238 | 0.545 | 0.959 |
| Cortical Thickness Average of Right Parahippocampal | rs1532278 | 27466315 | 238 | 0.912 | 0.959 |
| Cortical Thickness Average of Right Parahippocampal | rs2279590 | 27456253 | 238 | 0.579 | 0.959 |
| Cortical Thickness Average of Right Parahippocampal | rs7012010 | 27448729 | 238 | 0.959 | 0.959 |
| Cortical Thickness Average of Right Parahippocampal | rs7982 | 27462481 | 238 | 0.769 | 0.959 |
| Cortical Thickness Average of Right Parahippocampal | rs9331888 | 27468862 | 237 | 0.831 | 0.959 |
| Cortical Thickness Average of Right Parahippocampal | rs9331949 | 27454686 | 235 | 0.568 | 0.959 |
| Cortical Thickness Average of Right Posterior Cingulate | rs11136000 | 27464519 | 238 | 0.133 | 0.329 |
| Cortical Thickness Average of Right Posterior Cingulate | rs1532278 | 27466315 | 238 | 0.141 | 0.329 |
| Cortical Thickness Average of Right Posterior Cingulate | rs2279590 | 27456253 | 238 | 0.133 | 0.329 |
| Cortical Thickness Average of Right Posterior Cingulate | rs7012010 | 27448729 | 238 | 0.843 | 0.843 |
| Cortical Thickness Average of Right Posterior Cingulate | rs7982 | 27462481 | 238 | 0.21 | 0.35 |
| Cortical Thickness Average of Right Posterior Cingulate | rs9331888 | 27468862 | 237 | 0.58 | 0.676 |
| Cortical Thickness Average of Right Posterior Cingulate | rs9331949 | 27454686 | 235 | 0.25 | 0.35 |
| Cortical Thickness Average of Right Precuneus | rs11136000 | 27464519 | 238 | 0.947 | 0.947 |
| Cortical Thickness Average of Right Precuneus | rs1532278 | 27466315 | 238 | 0.692 | 0.947 |
| Cortical Thickness Average of Right Precuneus | rs2279590 | 27456253 | 238 | 0.462 | 0.855 |
| Cortical Thickness Average of Right Precuneus | rs7012010 | 27448729 | 238 | 0.208 | 0.855 |
| Cortical Thickness Average of Right Precuneus | rs7982 | 27462481 | 238 | 0.821 | 0.947 |
| Cortical Thickness Average of Right Precuneus | rs9331888 | 27468862 | 237 | 0.489 | 0.855 |
| Cortical Thickness Average of Right Precuneus | rs9331949 | 27454686 | 235 | 0.285 | 0.855 |
| Cortical Thickness Average of Left Entorhinal | rs11136000 | 27464519 | 238 | 0.312 | 0.542 |
| Cortical Thickness Average of Left Entorhinal | rs1532278 | 27466315 | 238 | 0.2 | 0.542 |
| Cortical Thickness Average of Left Entorhinal | rs2279590 | 27456253 | 238 | 0.387 | 0.542 |
| Cortical Thickness Average of Left Entorhinal | rs7012010 | 27448729 | 238 | 0.913 | 0.913 |
| Cortical Thickness Average of Left Entorhinal | rs7982 | 27462481 | 238 | 0.348 | 0.542 |
| Cortical Thickness Average of Left Entorhinal | rs9331888 | 27468862 | 237 | 0.604 | 0.704 |
| Cortical Thickness Average of Left Entorhinal | rs9331949 | 27454686 | 235 | 0.134 | 0.542 |
| Cortical Thickness Average of Left Middle Temporal | rs11136000 | 27464519 | 238 | 0.766 | 0.974 |
| Cortical Thickness Average of Left Middle Temporal | rs1532278 | 27466315 | 238 | 0.844 | 0.974 |
| Cortical Thickness Average of Left Middle Temporal | rs2279590 | 27456253 | 238 | 0.801 | 0.974 |
| Cortical Thickness Average of Left Middle Temporal | rs7012010 | 27448729 | 238 | 0.778 | 0.974 |
| Cortical Thickness Average of Left Middle Temporal | rs7982 | 27462481 | 238 | 0.974 | 0.974 |
| Cortical Thickness Average of Left Middle Temporal | rs9331888 | 27468862 | 237 | 0.536 | 0.974 |
| Cortical Thickness Average of Left Middle Temporal | rs9331949 | 27454686 | 235 | 0.777 | 0.974 |
| Cortical Thickness Average of Left Parahippocampal | rs11136000 | 27464519 | 237 | 0.312 | 0.822 |
| Cortical Thickness Average of Left Parahippocampal | rs1532278 | 27466315 | 237 | 0.566 | 0.822 |
| Cortical Thickness Average of Left Parahippocampal | rs2279590 | 27456253 | 237 | 0.822 | 0.822 |
| Cortical Thickness Average of Left Parahippocampal | rs7012010 | 27448729 | 237 | 0.529 | 0.822 |
| Cortical Thickness Average of Left Parahippocampal | rs7982 | 27462481 | 237 | 0.422 | 0.822 |
| Cortical Thickness Average of Left Parahippocampal | rs9331888 | 27468862 | 236 | 0.712 | 0.822 |
| Cortical Thickness Average of Left Parahippocampal | rs9331949 | 27454686 | 234 | 0.597 | 0.822 |
| Cortical Thickness Average of Left Posterior Cingulate | rs11136000 | 27464519 | 238 | 0.446 | 0.728 |
| Cortical Thickness Average of Left Posterior Cingulate | rs1532278 | 27466315 | 238 | 0.529 | 0.728 |
| Cortical Thickness Average of Left Posterior Cingulate | rs2279590 | 27456253 | 238 | 0.624 | 0.728 |
| Cortical Thickness Average of Left Posterior Cingulate | rs7012010 | 27448729 | 238 | 0.381 | 0.728 |
| Cortical Thickness Average of Left Posterior Cingulate | rs7982 | 27462481 | 238 | 0.439 | 0.728 |
| Cortical Thickness Average of Left Posterior Cingulate | rs9331888 | 27468862 | 237 | 0.053 | 0.372 |
| Cortical Thickness Average of Left Posterior Cingulate | rs9331949 | 27454686 | 235 | 0.78 | 0.78 |
| Cortical Thickness Average of Left Precuneus | rs11136000 | 27464519 | 238 | 0.962 | 0.964 |
| Cortical Thickness Average of Left Precuneus | rs1532278 | 27466315 | 238 | 0.66 | 0.964 |
| Cortical Thickness Average of Left Precuneus | rs2279590 | 27456253 | 238 | 0.842 | 0.964 |
| Cortical Thickness Average of Left Precuneus | rs7012010 | 27448729 | 238 | 0.733 | 0.964 |
| Cortical Thickness Average of Left Precuneus | rs7982 | 27462481 | 238 | 0.646 | 0.964 |
| Cortical Thickness Average of Left Precuneus | rs9331888 | 27468862 | 237 | 0.964 | 0.964 |
| Cortical Thickness Average of Left Precuneus | rs9331949 | 27454686 | 235 | 0.872 | 0.964 |
| Cortical Thickness Average of Right Entorhinal | rs11136000 | 27464519 | 238 | 0.042 | 0.146 |
| Cortical Thickness Average of Right Entorhinal | rs1532278 | 27466315 | 238 | 0.123 | 0.216 |
| Cortical Thickness Average of Right Entorhinal | rs2279590 | 27456253 | 238 | 0.235 | 0.274 |
| Cortical Thickness Average of Right Entorhinal | rs7012010 | 27448729 | 238 | 0.969 | 0.969 |
| Cortical Thickness Average of Right Entorhinal | rs7982 | 27462481 | 238 | 0.068 | 0.159 |
| Cortical Thickness Average of Right Entorhinal | rs9331888 | 27468862 | 237 | 0.011 | 0.075 |
| Cortical Thickness Average of Right Entorhinal | rs9331949 | 27454686 | 235 | 0.209 | 0.274 |
| Cortical Thickness Average of Right Middle Temporal | rs11136000 | 27464519 | 238 | 0.668 | 0.846 |
| Cortical Thickness Average of Right Middle Temporal | rs1532278 | 27466315 | 238 | 0.846 | 0.846 |
| Cortical Thickness Average of Right Middle Temporal | rs2279590 | 27456253 | 238 | 0.746 | 0.846 |
| Cortical Thickness Average of Right Middle Temporal | rs7012010 | 27448729 | 238 | 0.597 | 0.846 |
| Cortical Thickness Average of Right Middle Temporal | rs7982 | 27462481 | 238 | 0.433 | 0.846 |
| Cortical Thickness Average of Right Middle Temporal | rs9331888 | 27468862 | 237 | 0.483 | 0.846 |
| Cortical Thickness Average of Right Middle Temporal | rs9331949 | 27454686 | 235 | 0.435 | 0.846 |

NOTE: Pc value is the P value after false discovery rate (FDR) correction.

**Table S12. Association between *CLU* genetic variants and CSF**

| Component | SNP | BP | N | P | Pc |
| --- | --- | --- | --- | --- | --- |
| aBeta | rs11136000 | 27464519 | 501 | 0.264 | 0.355 |
| aBeta | rs1532278 | 27466315 | 501 | 0.304 | 0.355 |
| aBeta | rs2279590 | 27456253 | 501 | 0.414 | 0.414 |
| aBeta | rs7012010 | 27448729 | 501 | 0.287 | 0.355 |
| aBeta | rs7982 | 27462481 | 501 | 0.242 | 0.355 |
| aBeta | rs9331888 | 27468862 | 499 | 0.125 | 0.355 |
| aBeta | rs9331949 | 27454686 | 498 | 0.104 | 0.355 |
| Ptau | rs11136000 | 27464519 | 501 | 0.216 | 0.704 |
| Ptau | rs1532278 | 27466315 | 501 | 0.503 | 0.704 |
| Ptau | rs2279590 | 27456253 | 501 | 0.488 | 0.704 |
| Ptau | rs7012010 | 27448729 | 501 | 0.789 | 0.921 |
| Ptau | rs7982 | 27462481 | 501 | 0.399 | 0.704 |
| Ptau | rs9331888 | 27468862 | 499 | 0.23 | 0.704 |
| Ptau | rs9331949 | 27454686 | 498 | 0.977 | 0.977 |
| tau | rs11136000 | 27464519 | 501 | 0.026 | 0.179 |
| tau | rs1532278 | 27466315 | 501 | 0.182 | 0.255 |
| tau | rs2279590 | 27456253 | 501 | 0.17 | 0.255 |
| tau | rs7012010 | 27448729 | 501 | 0.729 | 0.851 |
| tau | rs7982 | 27462481 | 501 | 0.054 | 0.191 |
| tau | rs9331888 | 27468862 | 499 | 0.124 | 0.255 |
| tau | rs9331949 | 27454686 | 498 | 0.886 | 0.886 |

NOTE: Pc value is the P value after false discovery rate (FDR) correction.

**Table S13. Association between *CLU* genetic variants and glucose metabolism on FDG-PET at baseline**

| Region | SNP | BP | N | P | Pc |
| --- | --- | --- | --- | --- | --- |
| angular_l | rs11136000 | 27464519 | 565 | 0.312 | 0.832 |
| angular_l | rs1532278 | 27466315 | 565 | 0.566 | 0.96 |
| angular_l | rs2279590 | 27456253 | 565 | 0.852 | 0.96 |
| angular_l | rs7012010 | 27448729 | 565 | 0.96 | 0.96 |
| angular_l | rs7982 | 27462481 | 565 | 0.357 | 0.832 |
| angular_l | rs9331888 | 27468862 | 563 | 0.054 | 0.38 |
| angular_l | rs9331949 | 27454686 | 562 | 0.96 | 0.96 |
| angular_r | rs11136000 | 27464519 | 565 | 0.269 | 0.935 |
| angular_r | rs1532278 | 27466315 | 565 | 0.695 | 0.958 |
| angular_r | rs2279590 | 27456253 | 565 | 0.821 | 0.958 |
| angular_r | rs7012010 | 27448729 | 565 | 0.995 | 0.995 |
| angular_r | rs7982 | 27462481 | 565 | 0.401 | 0.935 |
| angular_r | rs9331888 | 27468862 | 563 | 0.316 | 0.935 |
| angular_r | rs9331949 | 27454686 | 562 | 0.725 | 0.958 |
| cigulumpost_b | rs11136000 | 27464519 | 565 | 0.335 | 0.913 |
| cigulumpost_b | rs1532278 | 27466315 | 565 | 0.913 | 0.913 |
| cigulumpost_b | rs2279590 | 27456253 | 565 | 0.765 | 0.913 |
| cigulumpost_b | rs7012010 | 27448729 | 565 | 0.614 | 0.913 |
| cigulumpost_b | rs7982 | 27462481 | 565 | 0.408 | 0.913 |
| cigulumpost_b | rs9331888 | 27468862 | 563 | 0.14 | 0.913 |
| cigulumpost_b | rs9331949 | 27454686 | 562 | 0.821 | 0.913 |
| temporal_l | rs11136000 | 27464519 | 565 | 0.073 | 0.232 |
| temporal_l | rs1532278 | 27466315 | 565 | 0.115 | 0.232 |
| temporal_l | rs2279590 | 27456253 | 565 | 0.262 | 0.354 |
| temporal_l | rs7012010 | 27448729 | 565 | 0.303 | 0.354 |
| temporal_l | rs7982 | 27462481 | 565 | 0.095 | 0.232 |
| temporal_l | rs9331888 | 27468862 | 563 | 0.132 | 0.232 |
| temporal_l | rs9331949 | 27454686 | 562 | 0.507 | 0.507 |
| temporal_r | rs11136000 | 27464519 | 565 | 0.356 | 0.906 |
| temporal_r | rs1532278 | 27466315 | 565 | 0.809 | 0.906 |
| temporal_r | rs2279590 | 27456253 | 565 | 0.84 | 0.906 |
| temporal_r | rs7012010 | 27448729 | 565 | 0.906 | 0.906 |
| temporal_r | rs7982 | 27462481 | 565 | 0.55 | 0.906 |
| temporal_r | rs9331888 | 27468862 | 563 | 0.38 | 0.906 |
| temporal_r | rs9331949 | 27454686 | 562 | 0.518 | 0.906 |

NOTE:Pc value is the P value after false discovery rate (FDR) correction.

**Table S14. Association between *CLU* genetic variants and glucose metabolism on FDG-PET at two-year follow-up study**

| Region | SNP | BP | N | P | Pc |
| --- | --- | --- | --- | --- | --- |
| angular_l | rs11136000 | 27464519 | 312 | 0.408 | 0.957 |
| angular_l | rs1532278 | 27466315 | 312 | 0.73 | 0.957 |
| angular_l | rs2279590 | 27456253 | 312 | 0.901 | 0.957 |
| angular_l | rs7012010 | 27448729 | 312 | 0.049 | 0.34 |
| angular_l | rs7982 | 27462481 | 312 | 0.773 | 0.957 |
| angular_l | rs9331888 | 27468862 | 311 | 0.957 | 0.957 |
| angular_l | rs9331949 | 27454686 | 310 | 0.49 | 0.957 |
| angular_r | rs11136000 | 27464519 | 312 | 0.793 | 0.96 |
| angular_r | rs1532278 | 27466315 | 312 | 0.939 | 0.96 |
| angular_r | rs2279590 | 27456253 | 312 | 0.486 | 0.96 |
| angular_r | rs7012010 | 27448729 | 312 | 0.24 | 0.96 |
| angular_r | rs7982 | 27462481 | 312 | 0.887 | 0.96 |
| angular_r | rs9331888 | 27468862 | 311 | 0.96 | 0.96 |
| angular_r | rs9331949 | 27454686 | 310 | 0.552 | 0.96 |
| cigulumpost_b | rs11136000 | 27464519 | 312 | 0.862 | 0.963 |
| cigulumpost_b | rs1532278 | 27466315 | 312 | 0.963 | 0.963 |
| cigulumpost_b | rs2279590 | 27456253 | 312 | 0.536 | 0.963 |
| cigulumpost_b | rs7012010 | 27448729 | 312 | 0.075 | 0.525 |
| cigulumpost_b | rs7982 | 27462481 | 312 | 0.917 | 0.963 |
| cigulumpost_b | rs9331888 | 27468862 | 311 | 0.792 | 0.963 |
| cigulumpost_b | rs9331949 | 27454686 | 310 | 0.534 | 0.963 |
| temporal_l | rs11136000 | 27464519 | 312 | 0.234 | 0.612 |
| temporal_l | rs1532278 | 27466315 | 312 | 0.424 | 0.612 |
| temporal_l | rs2279590 | 27456253 | 312 | 0.696 | 0.696 |
| temporal_l | rs7012010 | 27448729 | 312 | 0.086 | 0.601 |
| temporal_l | rs7982 | 27462481 | 312 | 0.438 | 0.612 |
| temporal_l | rs9331888 | 27468862 | 311 | 0.69 | 0.696 |
| temporal_l | rs9331949 | 27454686 | 310 | 0.308 | 0.612 |
| temporal_r | rs11136000 | 27464519 | 312 | 0.768 | 0.998 |
| temporal_r | rs1532278 | 27466315 | 312 | 0.815 | 0.998 |
| temporal_r | rs2279590 | 27456253 | 312 | 0.929 | 0.998 |
| temporal_r | rs7012010 | 27448729 | 312 | 0.176 | 0.998 |
| temporal_r | rs7982 | 27462481 | 312 | 0.829 | 0.998 |
| temporal_r | rs9331888 | 27468862 | 311 | 0.998 | 0.998 |
| temporal_r | rs9331949 | 27454686 | 310 | 0.457 | 0.998 |

NOTE:Pc value is the P value after false discovery rate (FDR) correction.

**Table S15. Association between *CLU* haplotypes and Aβ accumulation on AV45 at baseline**

| Region | Haplotype | P |
| --- | --- | --- |
| summarysuvr_composite_refnorm | GCCG | 0.0174 |
| summarysuvr_wholecerebnorm | ATTC | 0.0222 |
| summarysuvr_composite_refnorm | ATCC | 0.0257 |
| frontal | ATTC | 0.0278 |
| summarysuvr_composite_refnorm | ATTC | 0.0305 |
| cingulate | ATTC | 0.0324 |

**Figure S1. Linkage disequilibrium (LD) plot in the *CLU* SNPs**


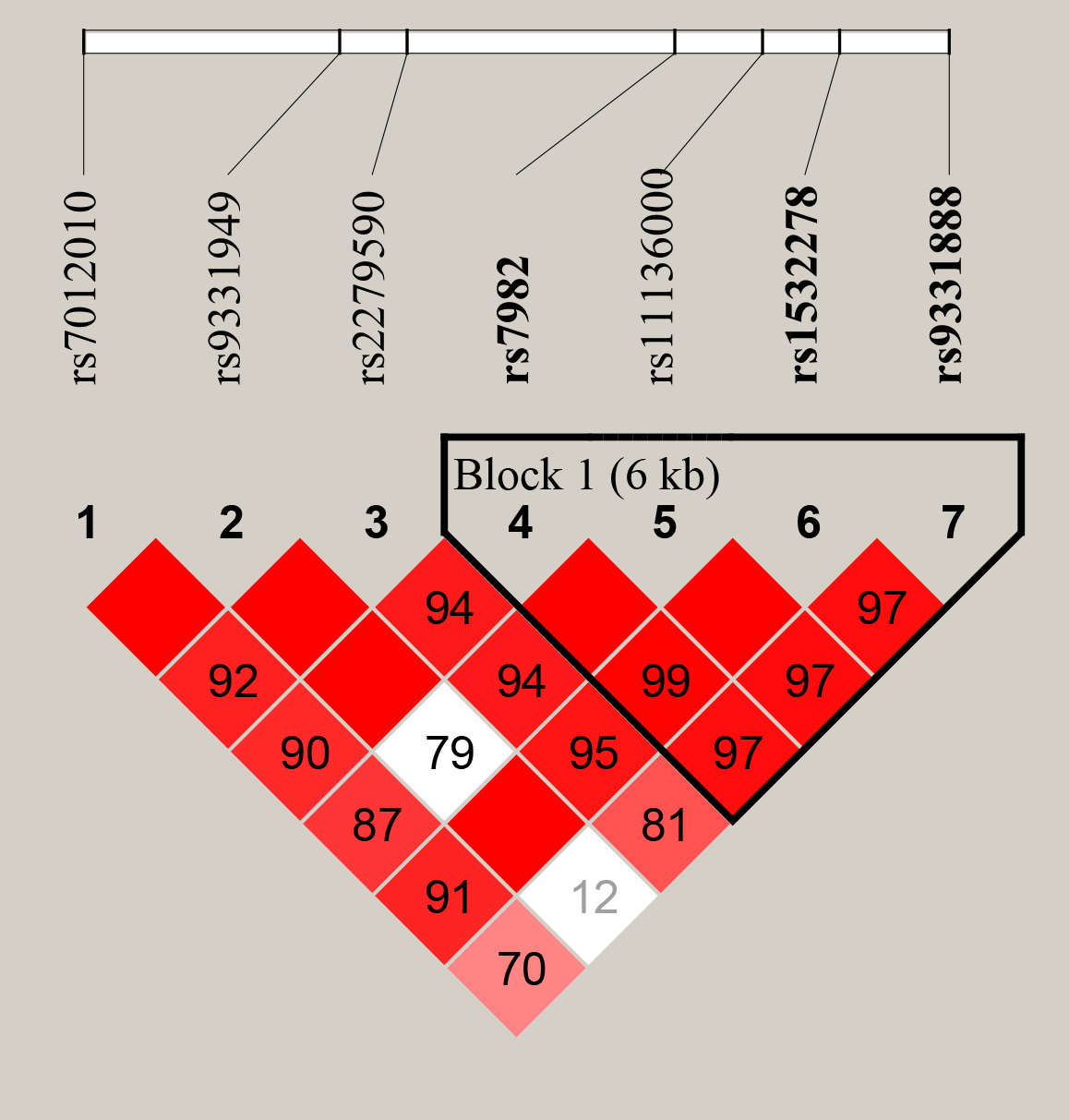


Note: This figure was drawn from the Haploview software.
